# Author Correction: Early methionine availability attenuates T cell exhaustion

**DOI:** 10.1038/s41590-026-02534-2

**Published:** 2026-05-07

**Authors:** Piyush Sharma, Ao Guo, Suresh Poudel, Emilio Boada-Romero, Katherine C. Verbist, Gustavo Palacios, Kalyan Immadisetty, Mark J. Chen, Dalia Haydar, Ashutosh Mishra, Junmin Peng, M. Madan Babu, Giedre Krenciute, Evan S. Glazer, Douglas R. Green

**Affiliations:** 1 Department of Immunology, St. Jude Children’s Research Hospital, Memphis, TN USA; 2 Department of Oncology, National Key Laboratory of Immune Response and Immunotherapy, The First Affiliated Hospital of USTC, Center for Advanced Interdisciplinary Science and Biomedicine of IHM, School of Basic Medical Sciences, Division of Life Sciences and Medicine, University of Science and Technology of China, Hefei, China; 3 Department of Structural Biology, Center of Excellence in Data Driven Discovery, St. Jude Children’s Research Hospital, Memphis, TN USA; 4 Department of Laboratory Medicine, Cleveland Clinic, Cleveland, OH USA; 5 Department of Bone Marrow Transplantation and Cellular Therapy, St. Jude Children’s Research Hospital, Memphis, TN USA; 6 Center for Proteomics and Metabolomics, St. Jude Children’s Research Hospital, Memphis, TN USA; 7 Department of Structural Biology, St. Jude Children’s Research Hospital, Memphis, TN USA; 8 Department of Surgery, University of Tennessee Health Science Center, Memphis, TN USA

**Keywords:** Tumour immunology, Translational immunology, T cells

Correction to: *Nature Immunology* 10.1038/s41590-025-02223-6, published online 23 July 2025.

In the version of the article initially published, the *x*-axis label in the right panel of Fig. 4d was “0.1 mM versus 0 mM” but should have been “0.1 mM versus 0.03 mM”. The original survival graph in Fig. 5j was incorrect and corresponded to a different dataset. Fig. 5j has now been corrected, as seen in Fig. 1, below. In Extended Data Fig. 10e, the *y*-axis label was missing and has now been added. These corrections have been made to the HTML and PDF versions of the article.

**Fig. 1:**
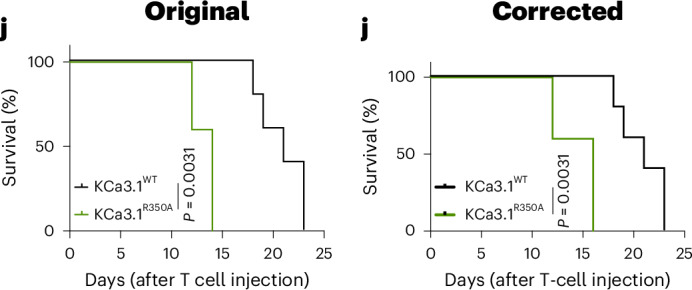
**Original and corrected Fig. 5j.**

