# Supplementary material for: Early methionine availability attenuates T cell exhaustion
Source: Nat Immunol. 2025 Jul 23;26(8):1384–96. doi: 10.1038/s41590-025-02223-6 (PMC12307228; doi:10.1038/s41590-025-02223-6)
Supplement: Supplementary file 1 — Reporting Summary [file 41590_2025_2223_MOESM1_ESM.pdf]

nature portfolio

Corresponding author(s): Douglas R. Green
Last updated by author(s): May 31, 2025

Reporting Summary

Nature Portfolio wishes to improve the reproducibility of the work that we publish. This form provides structure for consistency and transparency in reporting. For further information on Nature Portfolio policies, see our Editorial Policies and the Editorial Policy Checklist.

Statistics

For all statistical analyses, confirm that the following items are present in the figure legend, table legend, main text, or Methods section.

n/a	Confirmed	
☐	☒	The exact sample size (*n*) for each experimental group/condition, given as a discrete number and unit of measurement
☐	☒	A statement on whether measurements were taken from distinct samples or whether the same sample was measured repeatedly
☐	☒	The statistical test(s) used AND whether they are one- or two-sided<br>*Only common tests should be described solely by name; describe more complex techniques in the Methods section.*
☐	☒	A description of all covariates tested
☐	☒	A description of any assumptions or corrections, such as tests of normality and adjustment for multiple comparisons
☐	☒	A full description of the statistical parameters including central tendency (e.g. means) or other basic estimates (e.g. regression coefficient) AND variation (e.g. standard deviation) or associated estimates of uncertainty (e.g. confidence intervals)
☐	☒	For null hypothesis testing, the test statistic (e.g. *F*, *t*, *r*) with confidence intervals, effect sizes, degrees of freedom and *P* value noted<br>*Give P values as exact values whenever suitable.*
☒	☐	For Bayesian analysis, information on the choice of priors and Markov chain Monte Carlo settings
☒	☐	For hierarchical and complex designs, identification of the appropriate level for tests and full reporting of outcomes
☒	☐	Estimates of effect sizes (e.g. Cohen's *d*, Pearson's *r*), indicating how they were calculated

Our web collection on statistics for biologists contains articles on many of the points above.

Software and code

Policy information about availability of computer code

Data collection	BD FACSDiva software (LSRII and Fortessa) or SpectroFlo (Cytek Aurora)was used to collect flow cytometry data. Slidebook 6(3i) was used to collect confocal microscopy data. Bio-Rad ChemiDoc was used for immunoblot data acquisition.
Data analysis	Flowjo 10.8.2 for FACS; GraphPad 9.5.0 for statistics; slidebook 6 was used for microscope images; Affymetrix Expression Console v1.1; limma v.3.34.9 ; ggplot2 (v2.2.1); Trim Galore (version 0.5.0) ; Bowtie 2 (version 2.3.5.1) ; Picard MarkDuplicates function (version 2.19.0); SAMtools (version 1.9); BamTools (version 2.5.1); MACS (version 2.1.2); BEDTools (version 2.27.1); bedGraphToBigWig (version 377); deepTools plotHeatmap (version 3.2.1); DiffBind (version 2.16.0); ChIPseeker (version 1.26.2); clusterProfiler (version 3.18.1); STAR (version 2.7.5a) for CUT&RUN, ATAC-Seq and sequencing data.

For manuscripts utilizing custom algorithms or software that are central to the research but not yet described in published literature, software must be made available to editors and reviewers. We strongly encourage code deposition in a community repository (e.g. GitHub). See the Nature Portfolio guidelines for submitting code & software for further information.

March 2021

Data

Policy information about availability of data

All manuscripts must include a data availability statement. This statement should provide the following information, where applicable:

- Accession codes, unique identifiers, or web links for publicly available datasets
- A description of any restrictions on data availability
- For clinical datasets or third party data, please ensure that the statement adheres to our policy

RNA-seq, ATAC-seq and CUT&RUN data that support the findings of this study have been deposited in the Gene Expression Omnibus (GEO; https://www.ncbi.nlm.nih.gov/geo/) under accession number GSE299554, GSE299550 and GSE299551 respectively. Proteomics data has been deposited in PRIDE database with identifier number PXD064423. Publicly available database used in this study are from the Molecular Signatures Database (http://www.broadinstitute.org/gsea/msigdb/)

Human research participants

Policy information about studies involving human research participants and Sex and Gender in Research.

Reporting on sex and gender	Blood were from healthy blood donor. Age and other information of blood donor are unavailable.<br>Tumor samples were collected after surgery. Patients were de-indentified.
Population characteristics	No data on population characteristics was collected for this manuscript.
Recruitment	Blood from healthy normal blood donor were collected by the Blood Donor Center at St. Jude Children's Research Hospital. Apheresis rings for research are byproduct of normal donations. Samples were used without any selection.<br>For tumor samples, patients over 18 years of age were consented for their participation.
Ethics oversight	All human studies were in compliance with the Declaration of Helsinki. Blood donors were recruited by the Blood Donor Center at St. Jude Children's Research Hospital. Cancer patients were recruited by University of Tennessee Health Science Center. Blood donors provided written consent for research use of their blood products not used in transfusions and Cancer Patients provided written consent that tumor biopsies will be used for research purposes only, which has been reviewed and approved by the Institutional Review Board at St. Jude Children's Research Hospital and University of Tennessee Health Science Center.

Note that full information on the approval of the study protocol must also be provided in the manuscript.

Field-specific reporting

Please select the one below that is the best fit for your research. If you are not sure, read the appropriate sections before making your selection.

☒ Life sciences ☐ Behavioural & social sciences ☐ Ecological, evolutionary & environmental sciences

For a reference copy of the document with all sections, see nature.com/documents/nr-reporting-summary-flat.pdf

Life sciences study design

All studies must disclose on these points even when the disclosure is negative.

Sample size	No sample size calculation was performed to predetermine sample size. We determined the sample size based on similar experiments reports in previous publications (). Sample size was selected to maximize the chance of uncovering mean difference which is also statistically significant.
Data exclusions	One of the patient data was excluded according to the outlier test performed on Graphpad Prism. IN proteomics, one biological replicate M2 quantitative proteomics data showed higher variability (>SD), and therefore removed from the final differential expression analysis.
Replication	All the experimental finding were reproduced as validated by at least two independent experiments. For CUT&RUN experiment, at least two replicates were collected for each group. For amino acid LC-MS/MS measure, three independent biological replicates for group were analyzed.
Randomization	Age-and sex-matched mice were assigned randomly to experimental and control groups. For other experiments samples are randomly located into experiment groups.
Blinding	The investigators were not blinded to group allocation during data collection or analysis, as there was no subjective measurement in our experiments. This approach is considered standard for experiments of the type performed in this study.

Reporting for specific materials, systems and methods

We require information from authors about some types of materials, experimental systems and methods used in many studies. Here, indicate whether each material, system or method listed is relevant to your study. If you are not sure if a list item applies to your research, read the appropriate section before selecting a response.

Materials & experimental systems

n/a	Involved in the study
☐	☒ Antibodies
☐	☒ Eukaryotic cell lines
☒	☐ Palaeontology and archaeology
☐	☒ Animals and other organisms
☒	☐ Clinical data
☒	☐ Dual use research of concern

Methods

n/a	Involved in the study
☒	☐ ChIP-seq
☐	☒ Flow cytometry
☒	☐ MRI-based neuroimaging

Antibodies

Antibodies used

The following antibodies were used for flow cytometry:
Antibodies from BioLegend included Pacific Blue anti-mouse Ly108 (330-AJ, 134608), BV510 anti-mouse KLRG1 (2F1, 138421), BV570 anti-mouse CD62L (MEL-14, 104433), BV711 anti-mouse Tim-3 (RMT3-23, 119727), BV785 anti-mouse CD127 (A7R34, 135037), PE-Cy5 anti-mouse Granzyme-B (QA16A02, 372226), PE-Fire 700, anti-mouse CD4 (GK1.5, 100484), APC/Cy7 anti-mouse TNF-alpha (MP6-XT22, 506344), PE-Cy7 anti-mouse KLRG1 (2F1/KLRG1, 138416), PerCP-Cy 5.5 anti-mouse CD62L (MEL-14, 104432), BV605 anti-mouse CD127 (A7R34, 135025), Pacific blue anti-mouse CD69 (H1.2F3, 104524), Pacific blue anti-mouse CD45.1 (110722), APC anti-mouse PD1 (RL388, 109111), BV711 anti-mouse PD1 (29F.1A12, 135231), PE anti-mouse IFN-g (XMG1.2, 505808), FITC anti-mouse TNF-alpha(MP6-XT22, 506304), Pacific Blue anti-human CD45 (HI30, 982306), APC/Cy7 anti-human CD8 (RPA-T8, 344713). BUV563 anti-mouse LAG3 (C9B7W, 741350), BUV615 anti-mouse CD69 (H1.2F3, 751593), BUV805 anti-mouse CXCR3 (173, 748700), BV421 anti-mouse EOMES (X4-83, 567166), BV480 anti-mouse CD45.1 (A20, 746666), Alexa-Fluor 488 anti-mouse TCF1 (S33-966, 567018), Alexa-Fluor 647 TOX (NAN448B, 568356), BUV 496 anti-mouse Ly-108 (13G3, 750046), APC/Cy7 anti-mouse CD44 (IM7, 560568) and BUV805 anti-mouse CD8α (53-6.7, 612898) were acquired from BD Biosciences. BUV395 anti-mouse CD44 (IM7, 363-0441-82), PerCP-Cy 5.5 anti-mouse IL-2 (JES6-5H4, 45-7021-82), PerCP-eF710 anti-mouse CD27 (O323, 46-0279-42), and PE-Cy7 anti-mouse Tim3 (RMT3-23, 12-5870-82, eBioscience) were obtained from Thermo Fisher. APC anti-human/mouse Tox (REA473, 130-118-335) was obtained from Miltenyi Biotech.
All flow cytometry antibodies are used as 1:200.

The following antibodies were used for imaging:
anti-NFAT1 (1:250, cat. 4389, Cell Signaling Technology), anti-NFAT2 (1:250, D15F1, Cell Signaling Technology) and anti-mono and dimethyl arginine (1:500, cat. ab412, Abcam), anti-rabbit Alexa Fluor plus 595 (1:1,000, cat. A-11012, Thermo-Fisher), anti-mouse CD8-APC (1:500, cat. 100712, BioLegend), Alexa fluor 488 Phalloidin (1:1000, A12379, Thermo-Fisher) and Hoechst (1:1000, H3569, Thermo-Fisher).

The following antibodies were used for cell culture: anti-CD3 (145-2C11; Bio X Cell, BE0001) and anti-CD28 (37.51; Bio X Cell, BE0015-1).

Validation

The specificities of listed FACS antibodies have been validated by the manufacturer by flow cytometry.
Antibodies from BioLegend
Pacific Blue anti-mouse Ly108 (330-AJ, 134608); https://www.biolegend.com/en-us/products/pacific-blue-anti-mouse-ly108-antibody-6083?GroupID=BLG7404
BV510 anti-mouse KLRG1 (2F1, 138421); https://www.biolegend.com/en-us/products/brilliant-violet-510-anti-mouse-human-klrg1-mafa-antibody-9943
BV570 anti-mouse CD62L (MEL-14, 104433); https://www.biolegend.com/en-us/products/brilliant-violet-570-anti-mouse-cd62l-antibody-7369
BV711 anti-mouse Tim-3 (RMT3-23, 119727); https://www.biolegend.com/en-us/products/brilliant-violet-711-anti-mouse-cd366-tim-3-antibody-14918
BV785 anti-mouse CD127 (A7R34, 135037); https://www.biolegend.com/en-us/products/brilliant-violet-785-anti-mouse-cd127-il-7ralpha-antibody-10803
PE-Cy5 anti-mouse Granzyme-B (QA16A02, 372226); https://www.biolegend.com/en-us/products/pe-cyanine5-anti-human-mouse-granzyme-b-recombinant-antibody-21713
PE-Fire 700 anti-mouse CD4 (GK1.5, 100484); https://www.biolegend.com/en-us/products/pefire-700-anti-mouse-cd4-antibody-19781
APC/Cy7 anti-mouse TNF-alpha (MP6-XT22, 506344); https://www.biolegend.com/en-us/products/apc-cyanine7-anti-mouse-tnf-alpha-antibody-12117
PE-Cy7 anti-mouse KLRG1 (2F1/KLRG1, 138416); https://www.biolegend.com/en-us/products/pe-cyanine7-anti-mouse-human-klrg1-mafa-antibody-8312
PerCP-Cy 5.5 anti-mouse CD62L (MEL-14, 104432):https://www.biolegend.com/en-us/products/percp-cyanine5-5-anti-mouse-cd62l-antibody-4272?GroupID=BLG10534
BV605 anti-mouse CD127 (A7R34, 135025): https://www.biolegend.com/it-it/products/brilliant-violet-605-anti-mouse-cd127-il-7ralpha-antibody-8539

Pacific blue anti-mouse CD45.1(110722),;https://www.biolegend.com/en-us/products/pacific-blue-anti-mouse-cd45-1-antibody-3105
APC anti-mouse PD1 (RL388, 109111);https://www.biolegend.com/en-us/products/apc-anti-mouse-cd279-pd-1-antibody-6672?GroupID=BLG4702
BV711 anti-mouse PD1 (29F.1A12, 135231);https://www.biolegend.com/en-us/products/brilliant-violet-711-anti-mouse-cd279-pd-1-antibody-12303
PE anti-mouse IFN-g (XMG1.2, 505808);https://www.biolegend.com/en-us/products/pe-anti-mouse-ifn-gamma-antibody-997
FITC anti-mouse TNFa (MP6-XT22, 506304);https://www.biolegend.com/en-us/products/fitc-anti-mouse-tnf-alpha-antibody-976
Pacific Blue anti-human CD45 (HI30, 982306);https://www.biolegend.com/en-us/products/pacific-blue-anti-human-cd45-antibody-13991
APC/Cy7 anti-human CD8 (RPA-T8, 344713);https://www.biolegend.com/en-us/products/apc-cyanine7-anti-human-cd8-antibody-6391

Antibody from BD Biosciences
BUV563 anti-mouse LAG3 (C9B7W, 741350); https://www.bdbiosciences.com/en-us/products/reagents/flow-cytometry-reagents/research-reagents/single-color-antibodies-ruo/buv563-rat-anti-mouse-cd223.741350
BUV615 anti-mouse CD69 (H1.2F3, 751593); https://www.bdbiosciences.com/en-us/products/reagents/flow-cytometry-reagents/research-reagents/single-color-antibodies-ruo/buv615-hamster-anti-mouse-cd69.751593
BUV805 anti-mouse CXCR3 (173, 748700); https://www.bdbiosciences.com/en-us/products/reagents/flow-cytometry-reagents/research-reagents/single-color-antibodies-ruo/buv805-hamster-anti-mouse-cd183-cxcr3.748700
BV421 anti-mouse EOMES (X4-83, 567166); https://www.bdbiosciences.com/en-us/products/reagents/flow-cytometry-reagents/research-reagents/single-color-antibodies-ruo/bv421-mouse-anti-eomes.567166
BV480 anti-mouse CD45.1 (A20, 746666); https://www.bdbiosciences.com/en-us/products/reagents/flow-cytometry-reagents/research-reagents/single-color-antibodies-ruo/bv480-mouse-anti-mouse-cd45-1.746666
Alexa-Fluor 488 anti-mouse TCF1 (S33-966, 567018); https://www.bdbiosciences.com/en-us/products/reagents/flow-cytometry-reagents/research-reagents/single-color-antibodies-ruo/alexa-fluor-488-mouse-anti-tcf-7-tcf-1.567018
Alexa-Fluor 647 TOX (NAN448B, 568356);https://www.bdbiosciences.com/en-us/products/reagents/flow-cytometry-reagents/research-reagents/single-color-antibodies-ruo/alexa-fluor-647-rat-anti-tox.568356
BUV 496 anti-mouse Ly-108 (13G3, 750046);https://www.bdbiosciences.com/en-us/products/reagents/flow-cytometry-reagents/research-reagents/single-color-antibodies-ruo/buv496-mouse-anti-mouse-ly-108.750046
APC/Cy7 anti-mouse CD44 (IM7, 560568); https://www.bdbiosciences.com/en-us/products/reagents/flow-cytometry-reagents/research-reagents/single-color-antibodies-ruo/apc-cy-7-rat-anti-mouse-cd44.560568
BUV805 anti-mouse CD8α (53-6.7, 612898); https://www.bdbiosciences.com/en-us/products/reagents/flow-cytometry-reagents/research-reagents/single-color-antibodies-ruo/buv805-rat-anti-mouse-cd8a.612898

Antibody from Miltenyi Biotech
APC anti-human/mouse Tox (REA473, 130-118-335); https://www.miltenyibiotec.com/US-en/products/tox-antibody-anti-human-mouse-reafinity-rea473.html#apc:30-tests-in-60-ul

Antibody from Thermo-Fisher
PE-Cy7 anti-mouse Tim3 (RMT3-23, 12-5870-82); https://www.thermofisher.com/antibody/product/CD366-TIM3-Antibody-clone-RMT3-23-Monoclonal/12-5870-82

The specificities of listed imaging antibodies have been validated by the manufacturer by imaging.
anti-NFAT1 (cat. 4389, Cell Signaling Technology); https://www.cellsignal.com/products/primary-antibodies/nfat1-antibody/4389
anti-NFAT2 (cat. D15F1, Cell Signaling Technology); https://www.cellsignal.com/products/primary-antibodies/nfat2-d15f1-rabbit-mab/8032?site-search-type=Products&N=4294956287&Ntt=d15f1&fromPage=plp&_requestid=2192860
anti-mono and dimethyl arginine (cat. ab412, Abcam);https://www.abcam.com/mono-and-dimethyl-arginine-antibody-7e6-ab412.html
anti-rabbit Alexa Fluor plus 595 (cat. A-11012, Thermo-Fisher); https://www.thermofisher.com/antibody/product/Goat-anti-Rabbit-IgG-H-L-Cross-Adsorbed-Secondary-Antibody-Polyclonal/A-11012
Alexa fluor 488 Phalloidin (cat. A12379, Thermo-Fisher); https://www.thermofisher.com/order/catalog/product/A12379
Hoechst 33258 (cat. H3569, Thermo-Fisher); https://www.thermofisher.com/order/catalog/product/H3569

The listed antibodies have been validated by the manufacturer:
anti-CD3e (145-2C11; Bio X Cell, BE0001):https://bxcell.com/product/m-cd3e/
anti-CD28 (37.51; Bio X Cell, BE0015-1): https://bxcell.com/product/m-cd28/

Eukaryotic cell lines

Policy information about cell lines and Sex and Gender in Research

Cell line source(s)	B16-Ova was kindly provided by Hongbo Chi and MC38-Ova cell line were kindly provided by Dr. Dario Vignali. F420 cell line was provided by Dr. Jason T Yustein. Plat-e cells were purchased from Cell Biolabs (cat. RV-101).
Authentication	The cell line used was not authenticated
Mycoplasma contamination	B16-Ova, MC38, MC38-Ova, LLC, F420 were checked for mycoplasma contamination and found to be negative.
Commonly misidentified lines (See ICLAC register)	No commonly misidentified cell line were used.

Animals and other research organisms

Policy information about studies involving animals; ARRIVE guidelines recommended for reporting animal research, and Sex and Gender in Research

Laboratory animals	Mice were housed and bred at the St. Jude Children's Research Hospital Animal Resource Center in specific pathogen-free conditions. Mice were on 12-hour light/dark cycles that coincide with daylight in Memphis, TN, USA. The St. Jude Children's Research Hospital Animal Resource Center housing facility was maintained at 20–25 °C and 30–70 % humidity. All genetic models were on the C57BL/6 background. Both male and female mice were used for analysis and quantification. All mice were used at 6-10 weeks old.<br>We crossed Rosa26-Cas9 knock-in mice with OT-I/P14 transgenic mice to express Cas9 in antigen-specific CD8+ T cells (called Cas9-OT-I mice or Cas9-P14). The Cas9 mice were fully backcrossed to the C57BL/6J background. Rag1–/– mice were purchased from the Jackson Laboratory.
Wild animals	This study did not involve wild animals
Reporting on sex	Sex of the mice was not considered during experimental planning.
Field-collected samples	The study did not involve samples collected from the field.
Ethics oversight	Mouse studies were conducted in accordance with protocols approved by the St. Jude Children's Research Hospital Committee on Care and Use of Animals and in compliance with all relevant ethical guidelines.

Note that full information on the approval of the study protocol must also be provided in the manuscript.

Flow Cytometry

Plots

Confirm that:

☒ The axis labels state the marker and fluorochrome used (e.g. CD4-FITC).

☒ The axis scales are clearly visible. Include numbers along axes only for bottom left plot of group (a 'group' is an analysis of identical markers).

☒ All plots are contour plots with outliers or pseudocolor plots.

☒ A numerical value for number of cells or percentage (with statistics) is provided.

Methodology

Sample preparation	The spleens and peripheral lymph nodes (PLNs) were gently separated under nylon mesh using the flat end of a 3-mL syringes. Red blood cells were removed by ACK lysing buffer, followed by washing cells with isolation buffer. After spinning down, the cell pellets were resuspended and filtered with nylon mesh before staining.<br>For the examination of tumour infiltrating lymphocytes, tumours were excised, minced and digested with 0.5 mg/ml Collagenase IV (Roche) + 200 UI/ml DNase I (Sigma) for 40 min at 37 °C, and then passed through 70-µm filters to remove undigested tumor tissues.
Instrument	Fortessa (BD Bioscience) or Aurora (Cytek).
Software	Flowjo 10.8.2
Cell population abundance	For sorting CRISPR/Cas9 generated KCa3.1WT/R350A mutant, the population varied from 25-50%.
Gating strategy	Based on the pattern of FSC-A/SSC-A, cells in the lymphocyte gate were used for analysis of T cell subsets. Singlets were gated according to the pattern of FSC-H vs. FSC-A. Positive populations were determined by the specific antibodies, which were distinct from negative populations.

☒ Tick this box to confirm that a figure exemplifying the gating strategy is provided in the Supplementary Information.
