# Supplementary figures and images for: Early methionine availability attenuates T cell exhaustion
Source: Nat Immunol. 2025 Jul 23;26(8):1384–96. doi: 10.1038/s41590-025-02223-6 (PMC12307228; doi:10.1038/s41590-025-02223-6)

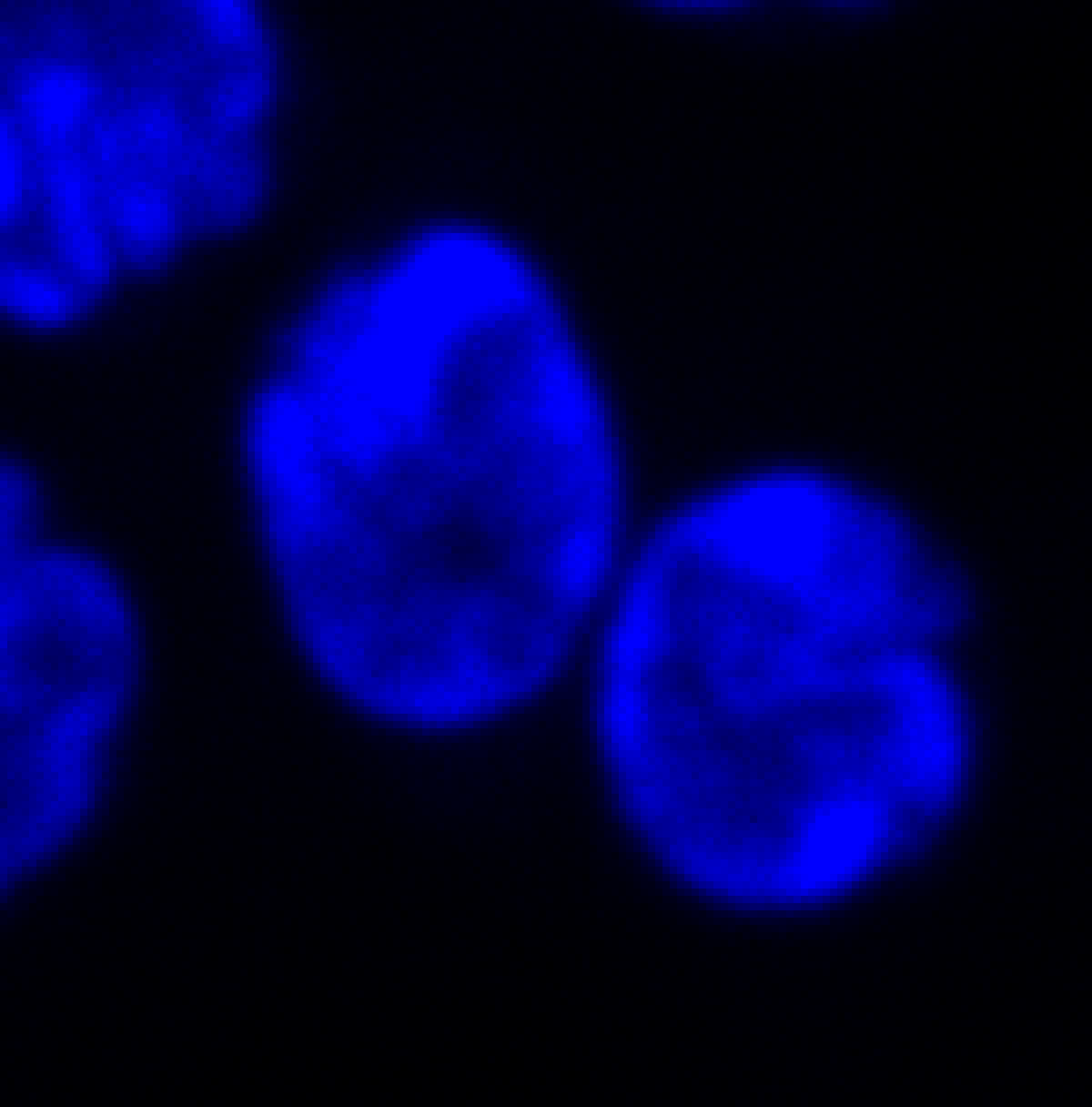

Supplement: Supplementary file 15 — Single images from Fig. 3d. [file 41590_2025_2223_MOESM15_ESM.zip › Sharma_Images_Fig3D/NAIVE HOECHST.tif]

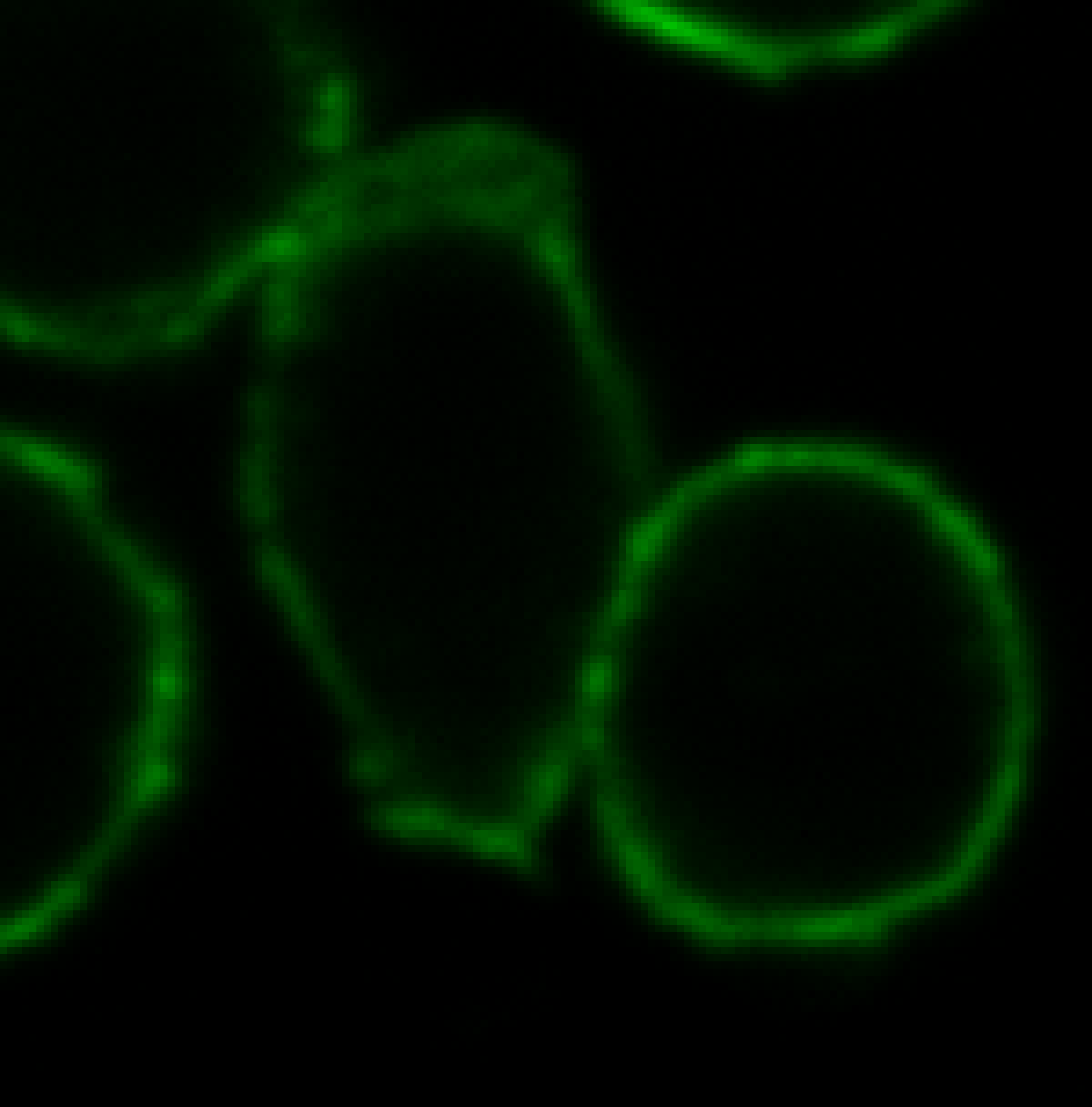

Supplement: Supplementary file 15 — Single images from Fig. 3d. [file 41590_2025_2223_MOESM15_ESM.zip › Sharma_Images_Fig3D/NAIVE PHALLOIDIN.tif]

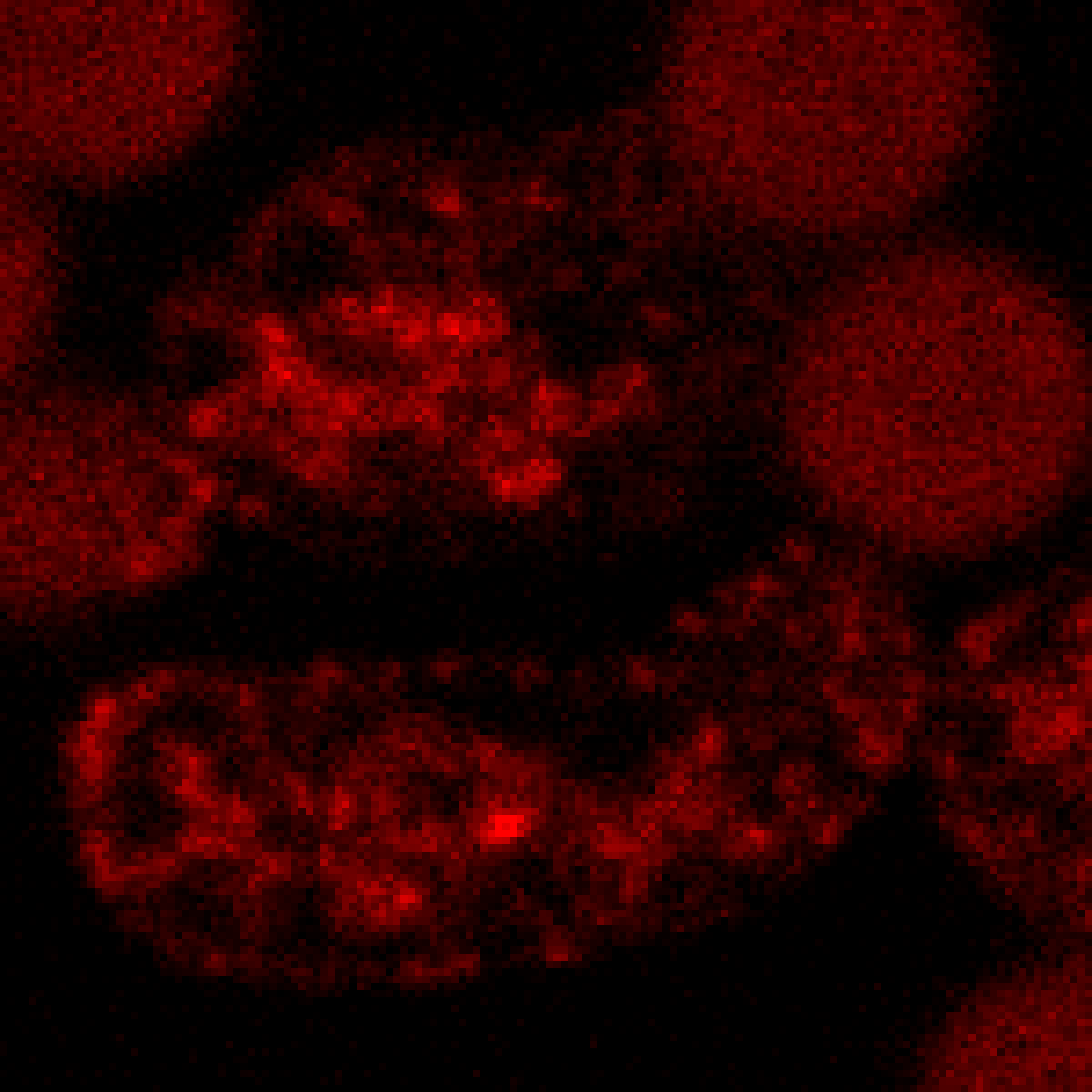

Supplement: Supplementary file 15 — Single images from Fig. 3d. [file 41590_2025_2223_MOESM15_ESM.zip › Sharma_Images_Fig3D/CONTROL 0.03mM NFAT1 .tif]

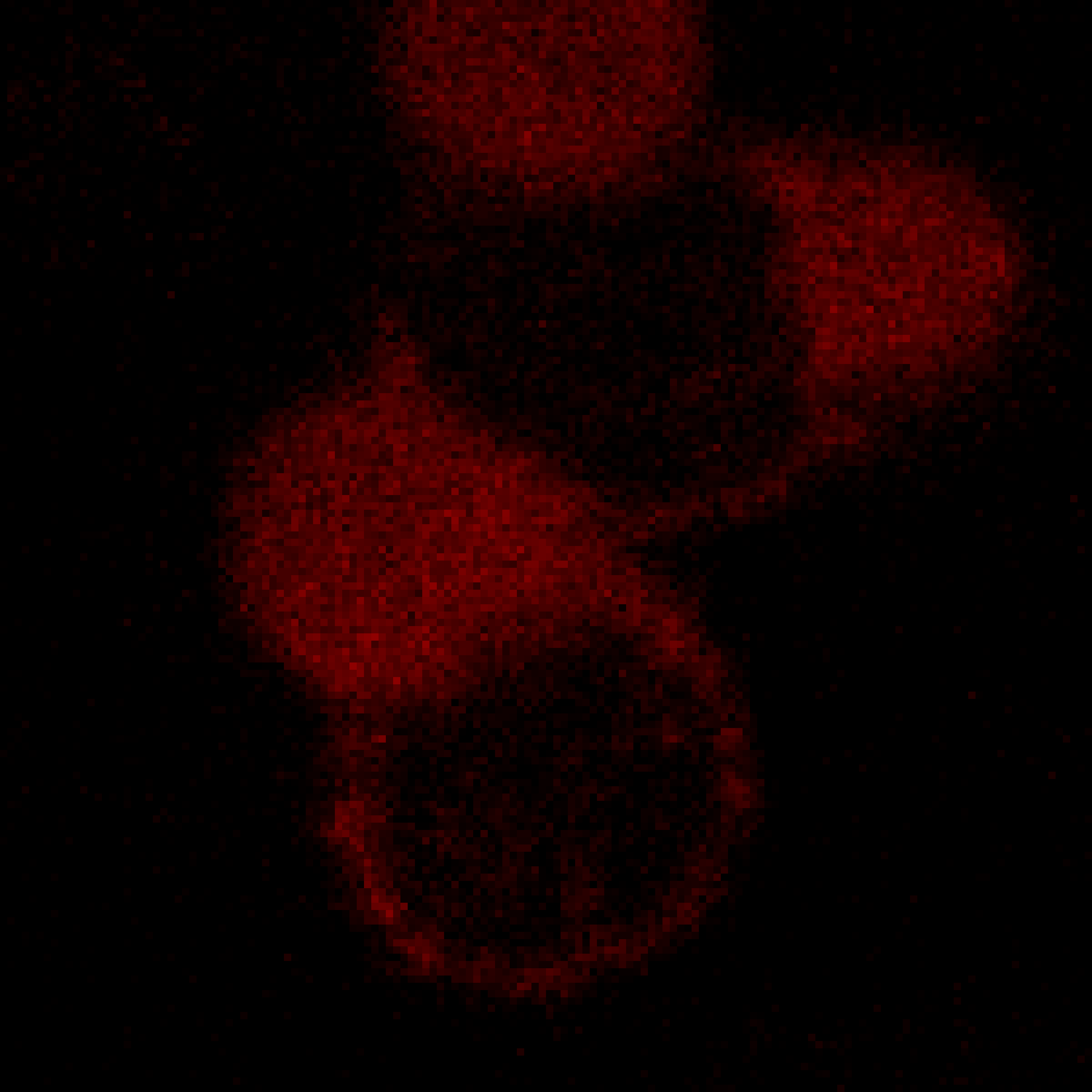

Supplement: Supplementary file 15 — Single images from Fig. 3d. [file 41590_2025_2223_MOESM15_ESM.zip › Sharma_Images_Fig3D/CsA 0.03mM NAFT1.tif]

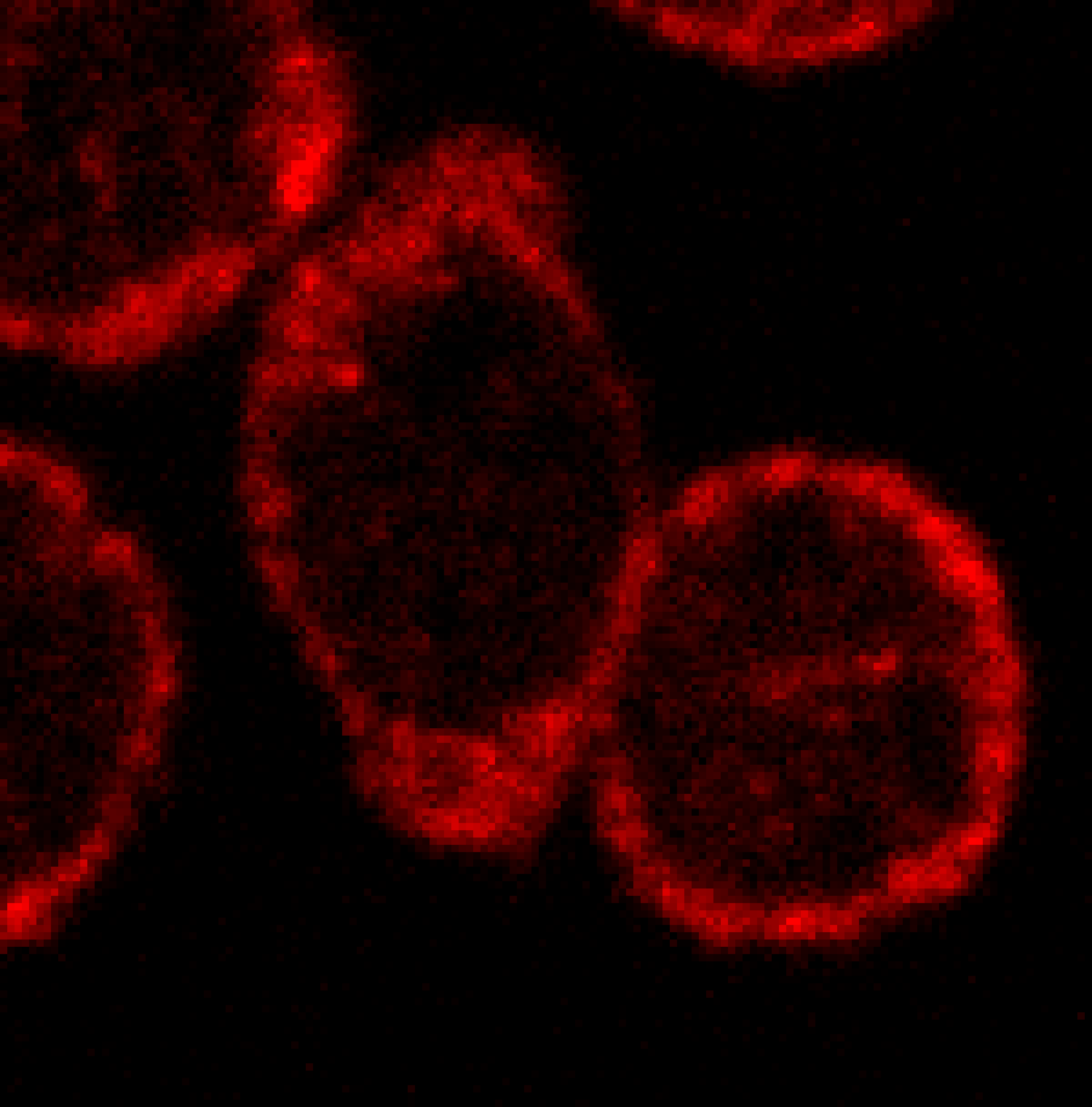

Supplement: Supplementary file 15 — Single images from Fig. 3d. [file 41590_2025_2223_MOESM15_ESM.zip › Sharma_Images_Fig3D/NAIVE NFAT1.tif]

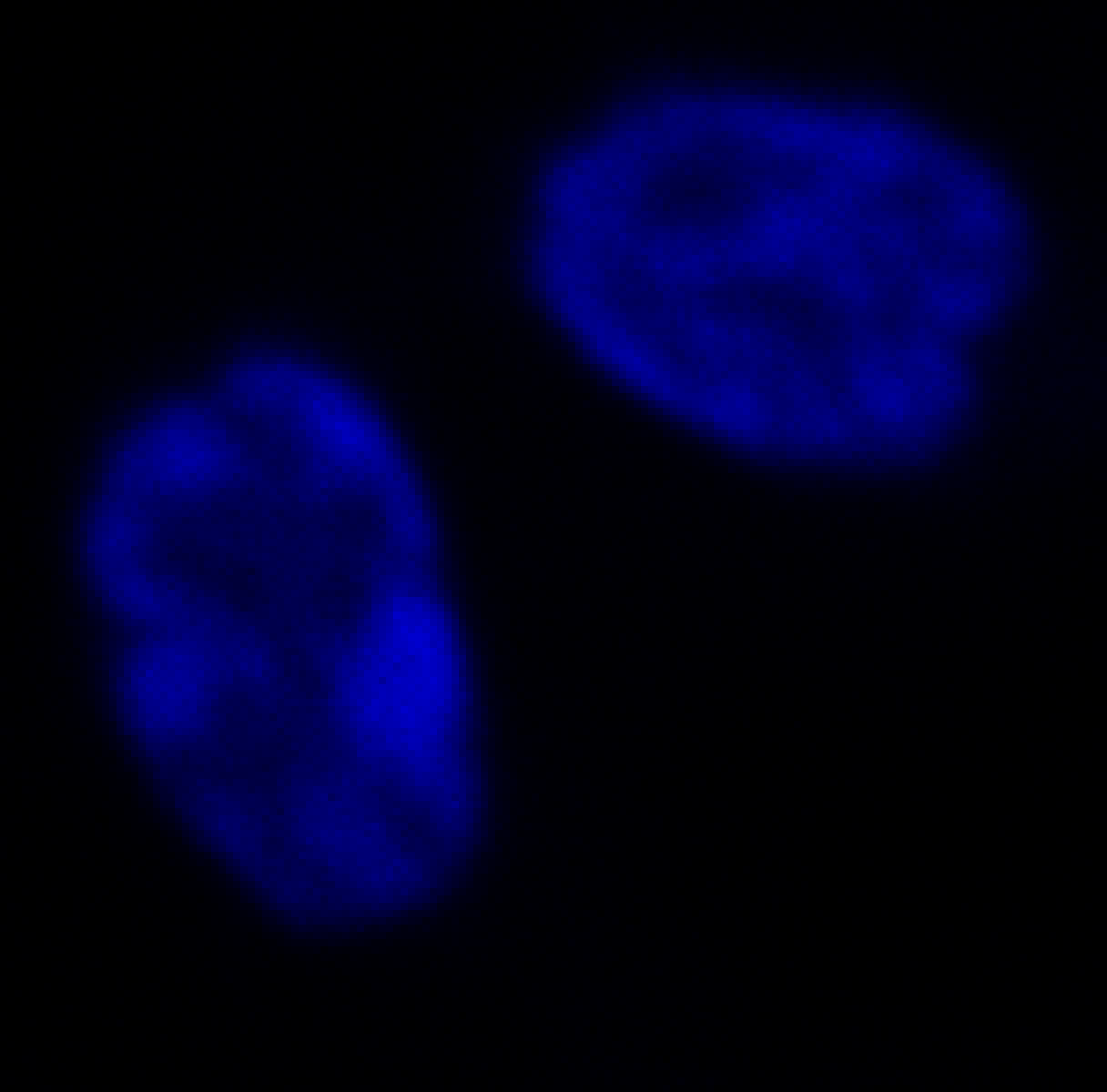

Supplement: Supplementary file 15 — Single images from Fig. 3d. [file 41590_2025_2223_MOESM15_ESM.zip › Sharma_Images_Fig3D/CONTROL 0.1mM HOECHST.tif]

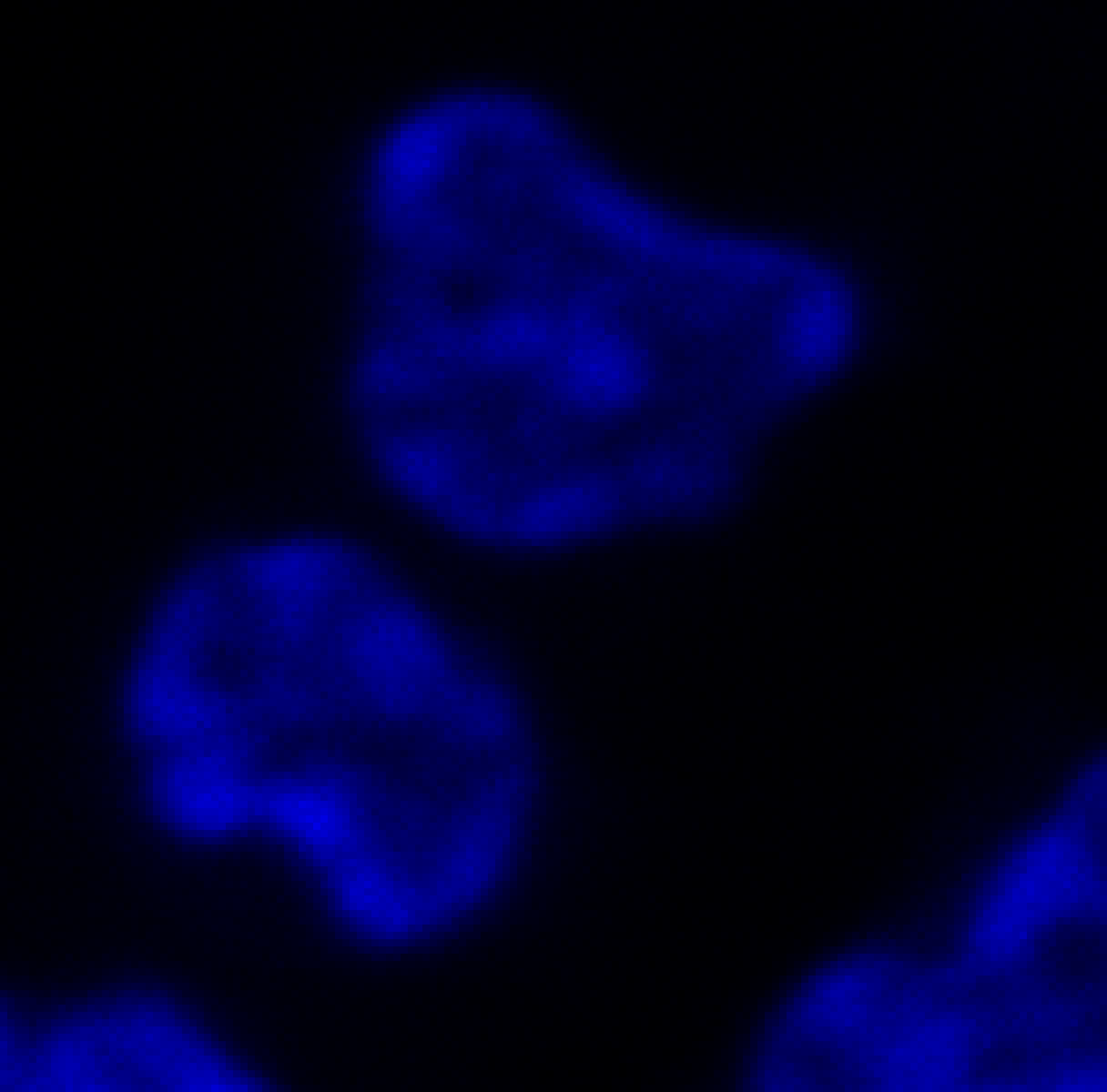

Supplement: Supplementary file 15 — Single images from Fig. 3d. [file 41590_2025_2223_MOESM15_ESM.zip › Sharma_Images_Fig3D/CsA 0.1mM HOECHST.tif]

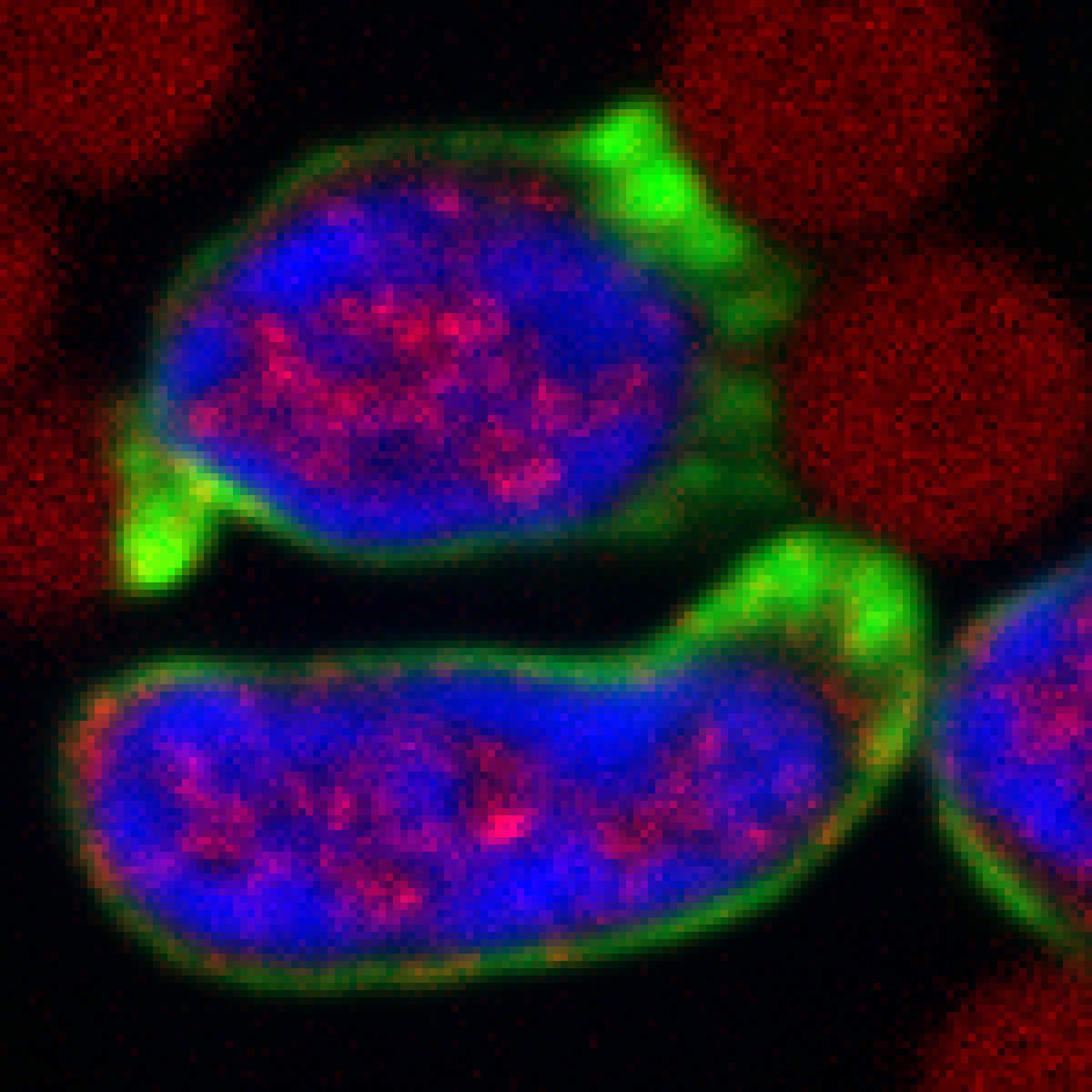

Supplement: Supplementary file 15 — Single images from Fig. 3d. [file 41590_2025_2223_MOESM15_ESM.zip › Sharma_Images_Fig3D/CONTROL 0.03mM MERGED .tif]

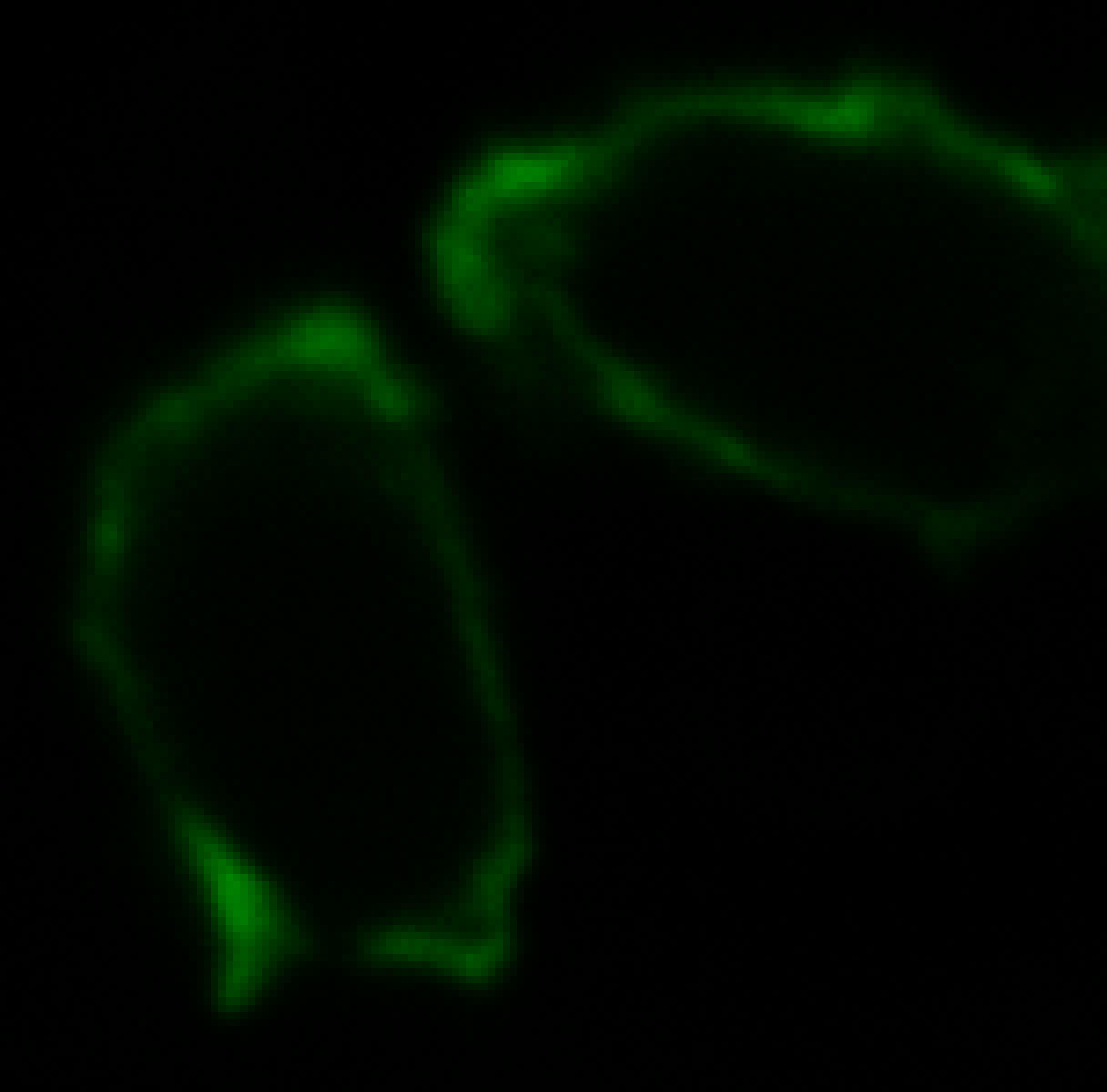

Supplement: Supplementary file 15 — Single images from Fig. 3d. [file 41590_2025_2223_MOESM15_ESM.zip › Sharma_Images_Fig3D/CONTROL 0.1mM PHALLOIDIN.tif]

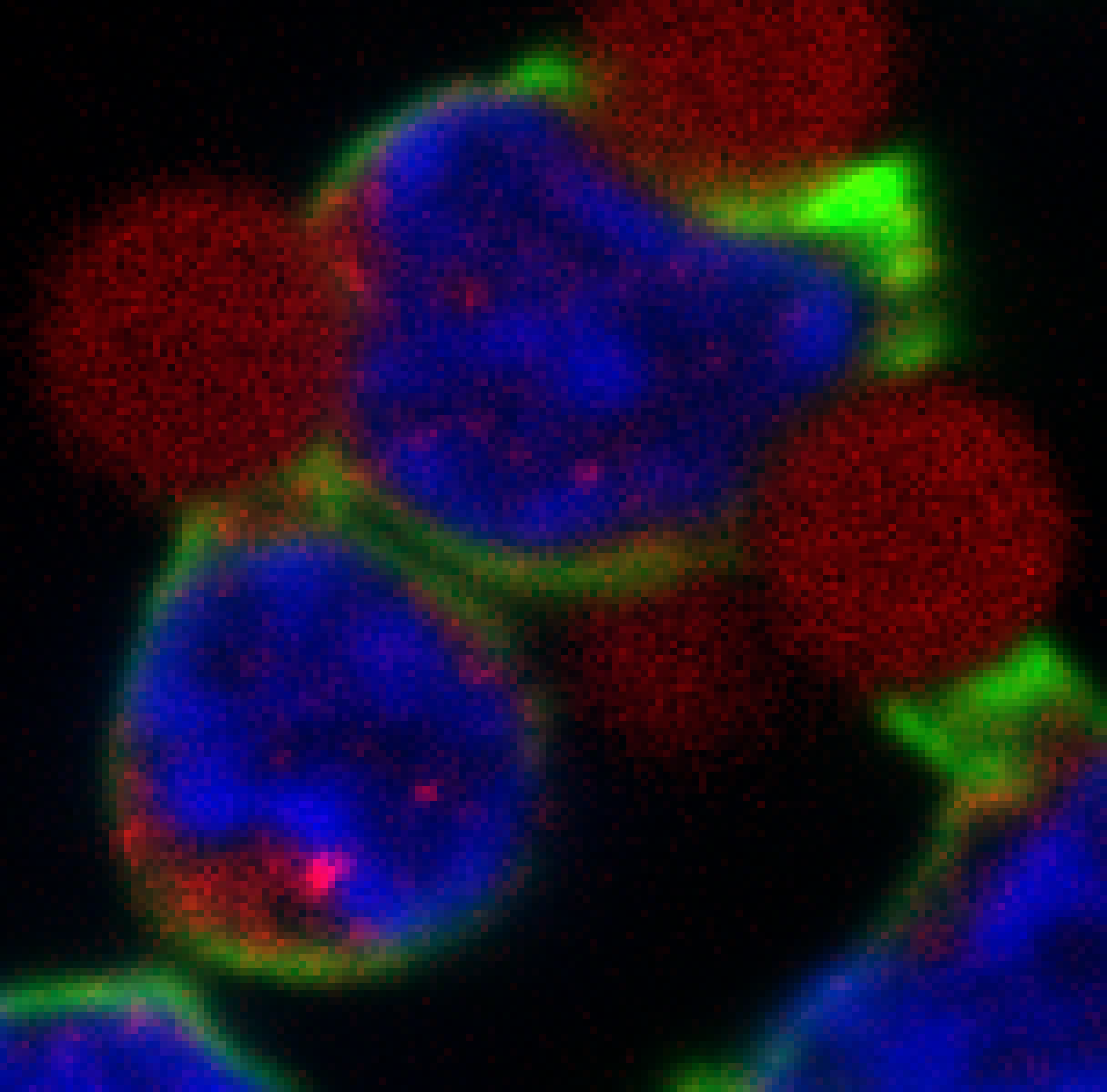

Supplement: Supplementary file 15 — Single images from Fig. 3d. [file 41590_2025_2223_MOESM15_ESM.zip › Sharma_Images_Fig3D/CsA 0.1mM MERGED.tif]

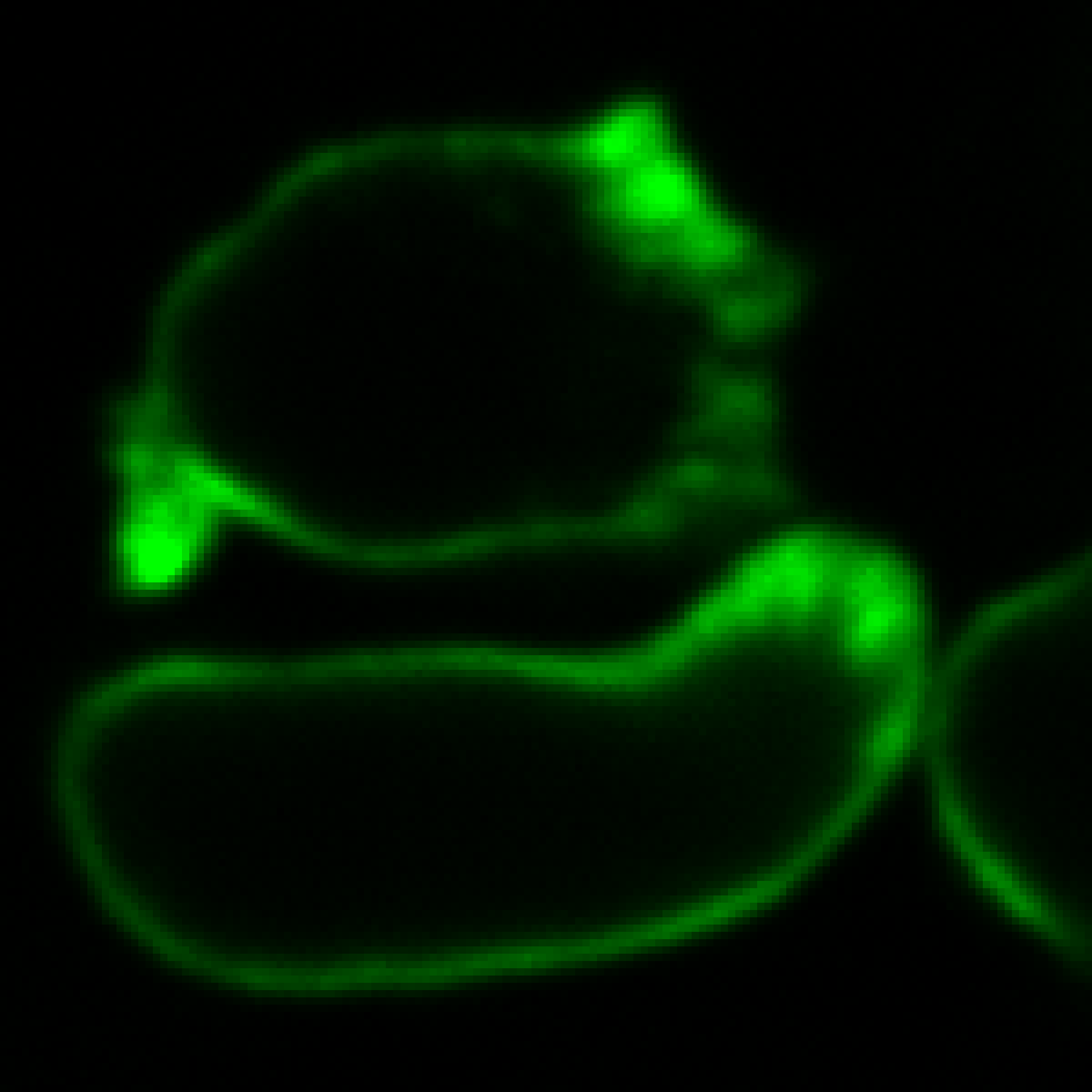

Supplement: Supplementary file 15 — Single images from Fig. 3d. [file 41590_2025_2223_MOESM15_ESM.zip › Sharma_Images_Fig3D/CONTROL 0.03mM PHALLOIDIN.tif]

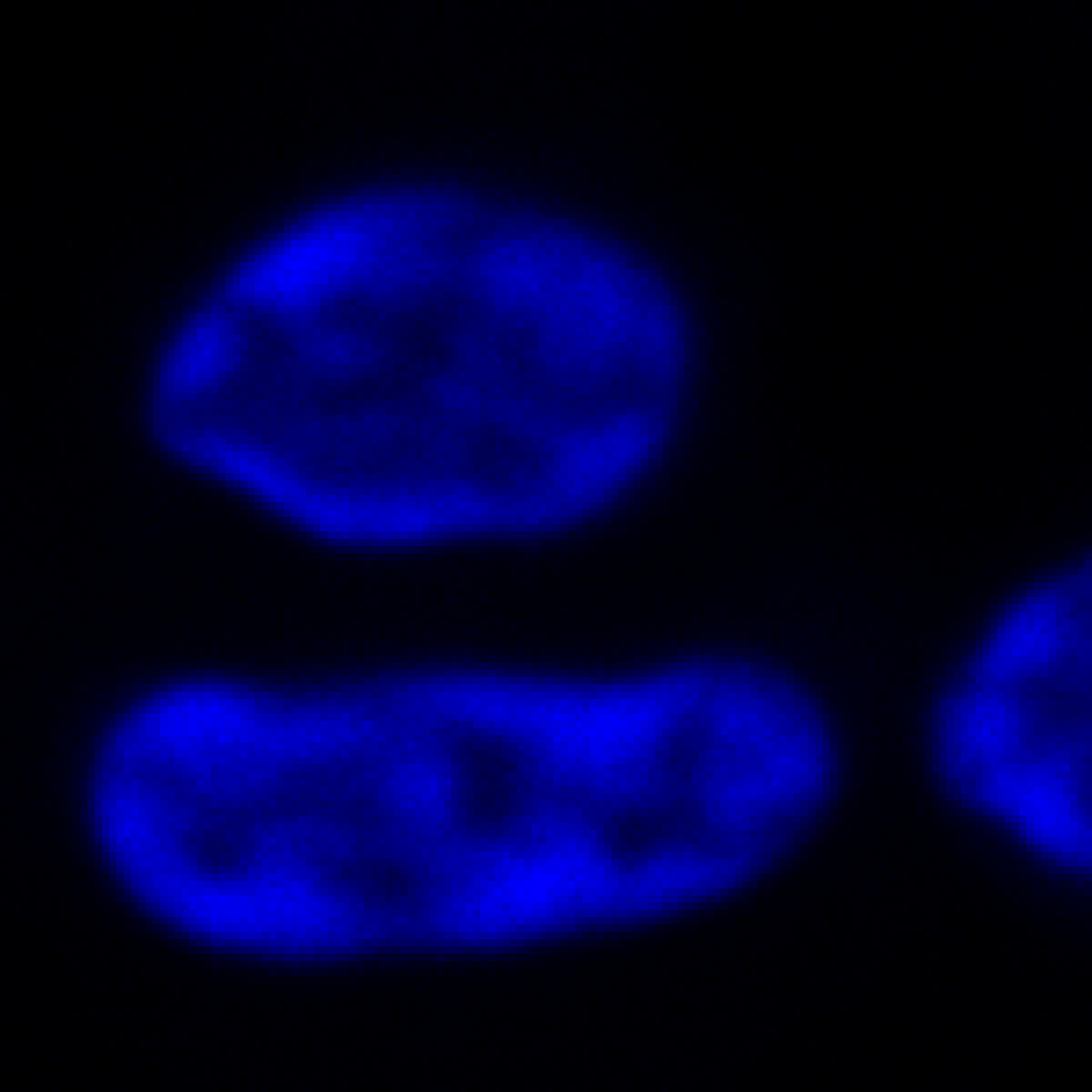

Supplement: Supplementary file 15 — Single images from Fig. 3d. [file 41590_2025_2223_MOESM15_ESM.zip › Sharma_Images_Fig3D/CONTROL 0.03mM HOECHST .tif]

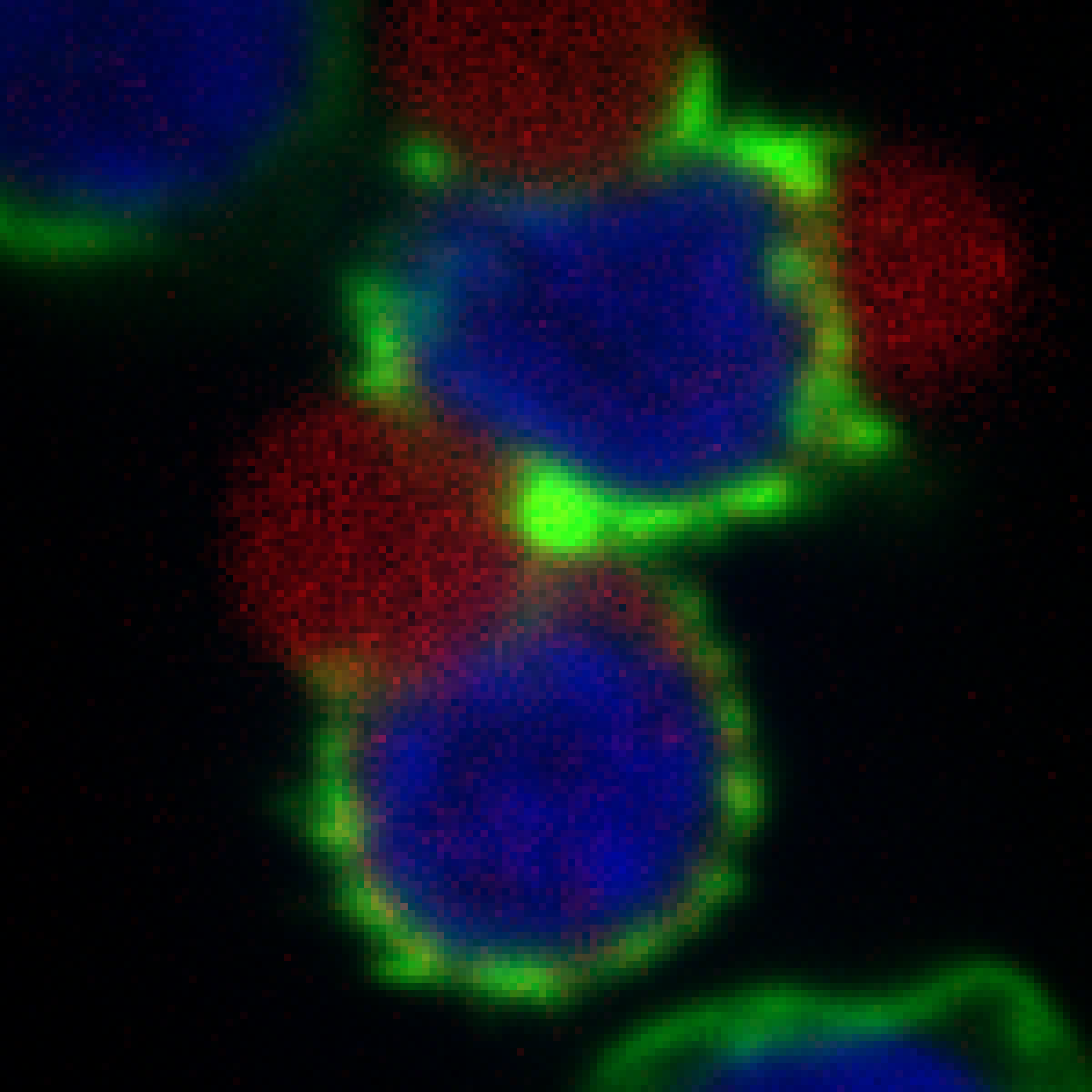

Supplement: Supplementary file 15 — Single images from Fig. 3d. [file 41590_2025_2223_MOESM15_ESM.zip › Sharma_Images_Fig3D/CsA 0.03mM MERGED.tif]

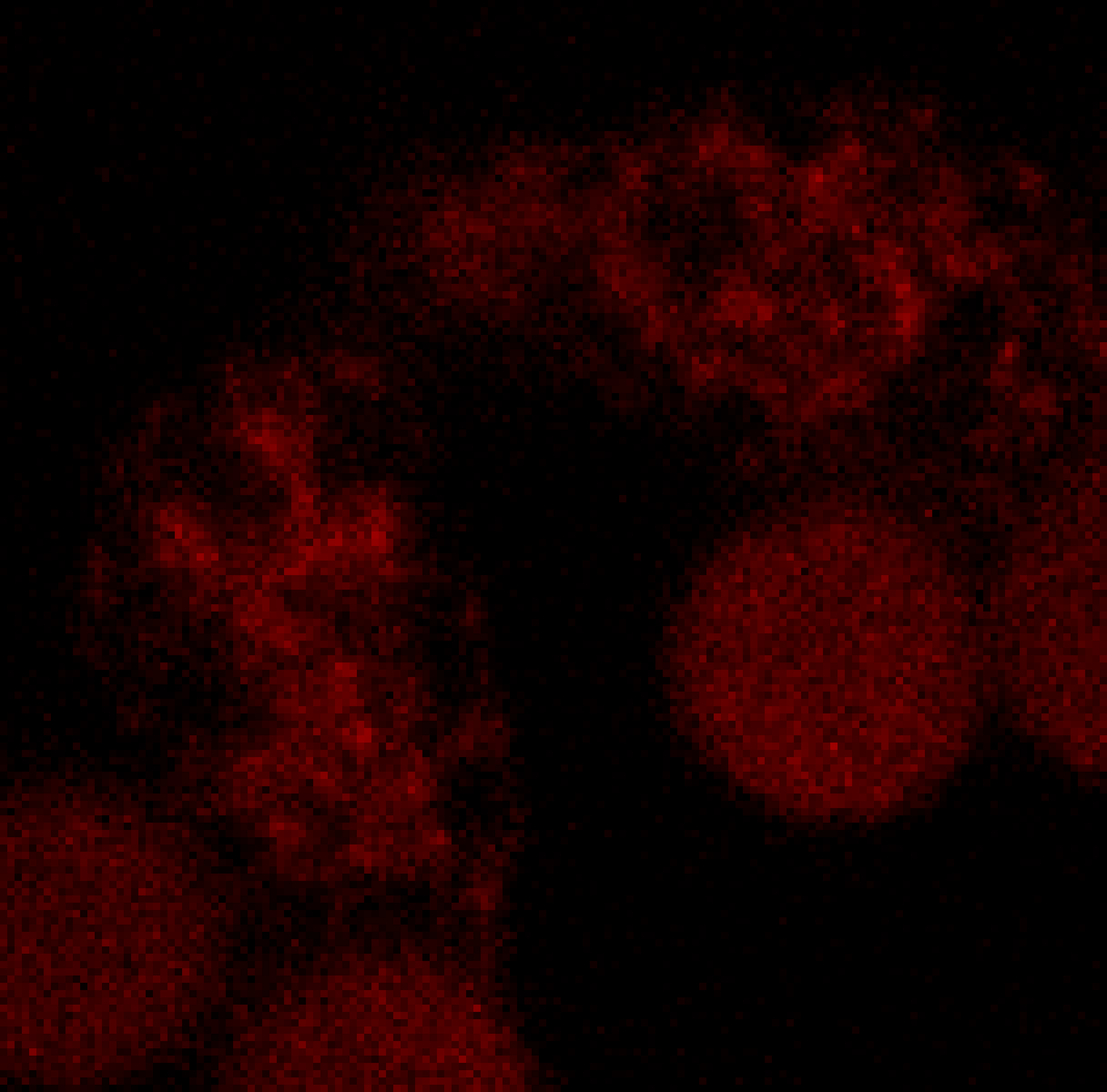

Supplement: Supplementary file 15 — Single images from Fig. 3d. [file 41590_2025_2223_MOESM15_ESM.zip › Sharma_Images_Fig3D/CONTROL 0.1mM NFAT1.tif]

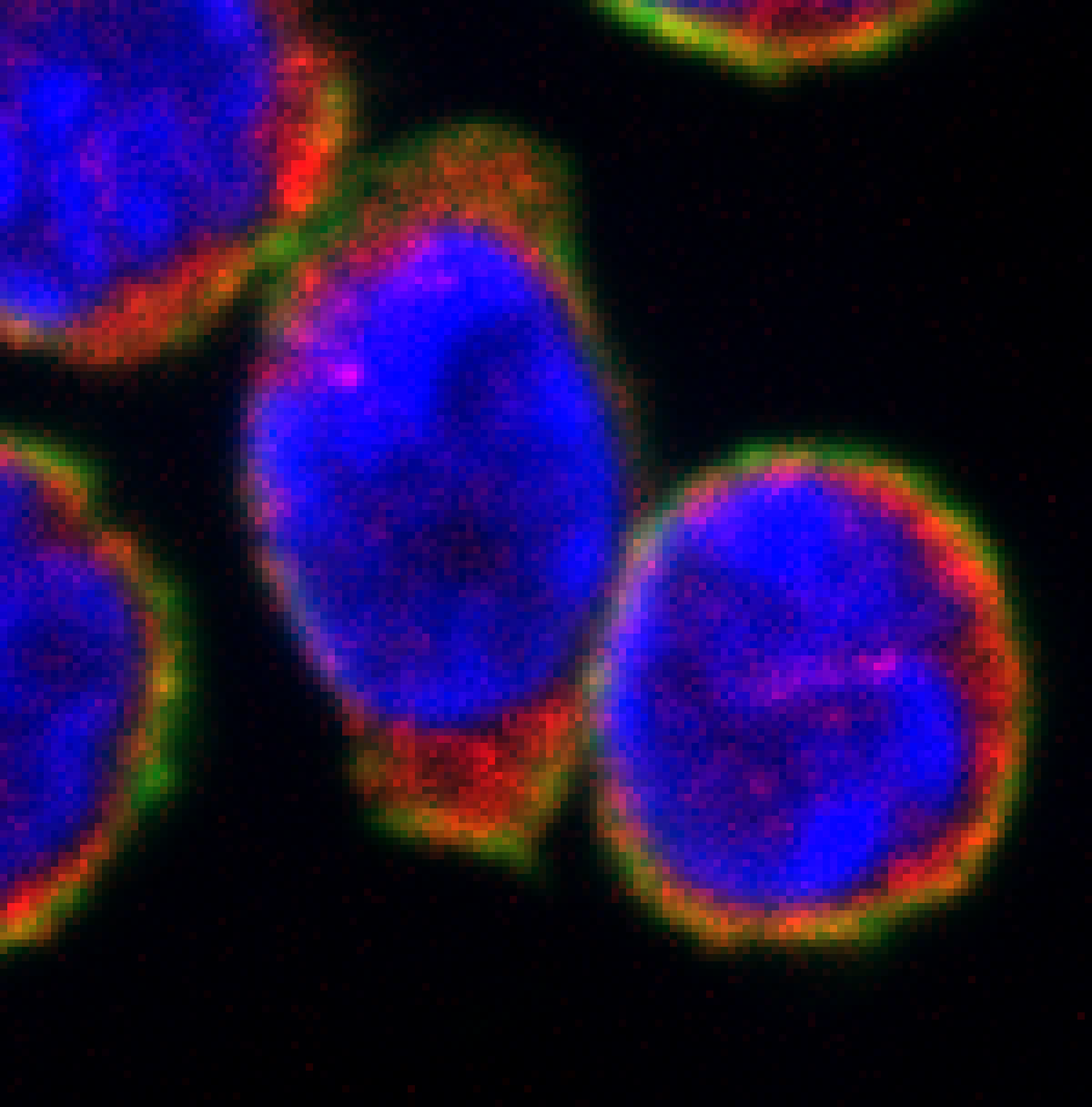

Supplement: Supplementary file 15 — Single images from Fig. 3d. [file 41590_2025_2223_MOESM15_ESM.zip › Sharma_Images_Fig3D/NAIVE MERGED.tif]

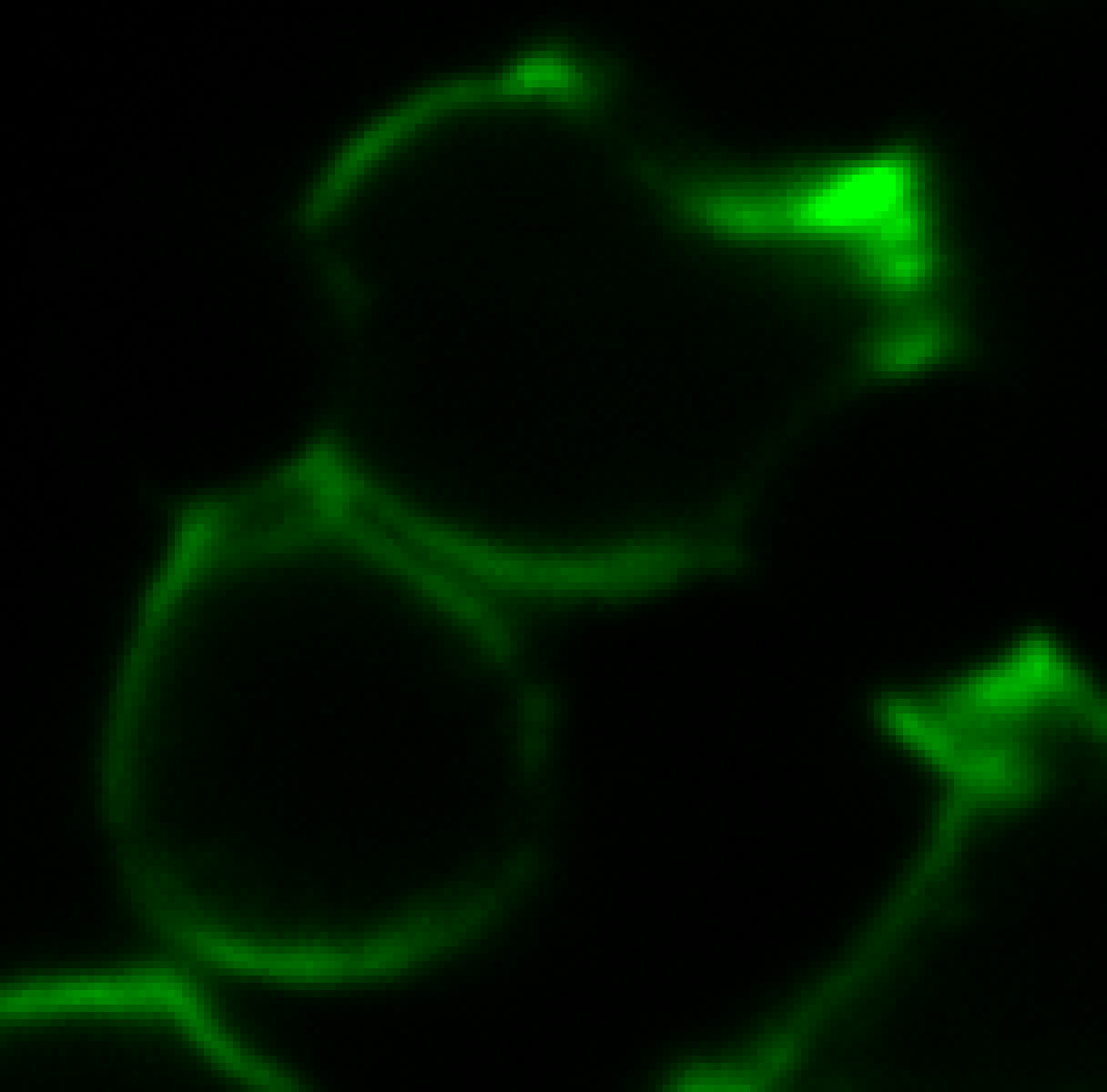

Supplement: Supplementary file 15 — Single images from Fig. 3d. [file 41590_2025_2223_MOESM15_ESM.zip › Sharma_Images_Fig3D/CsA 0.1mM PHALLOIDIN.tif]

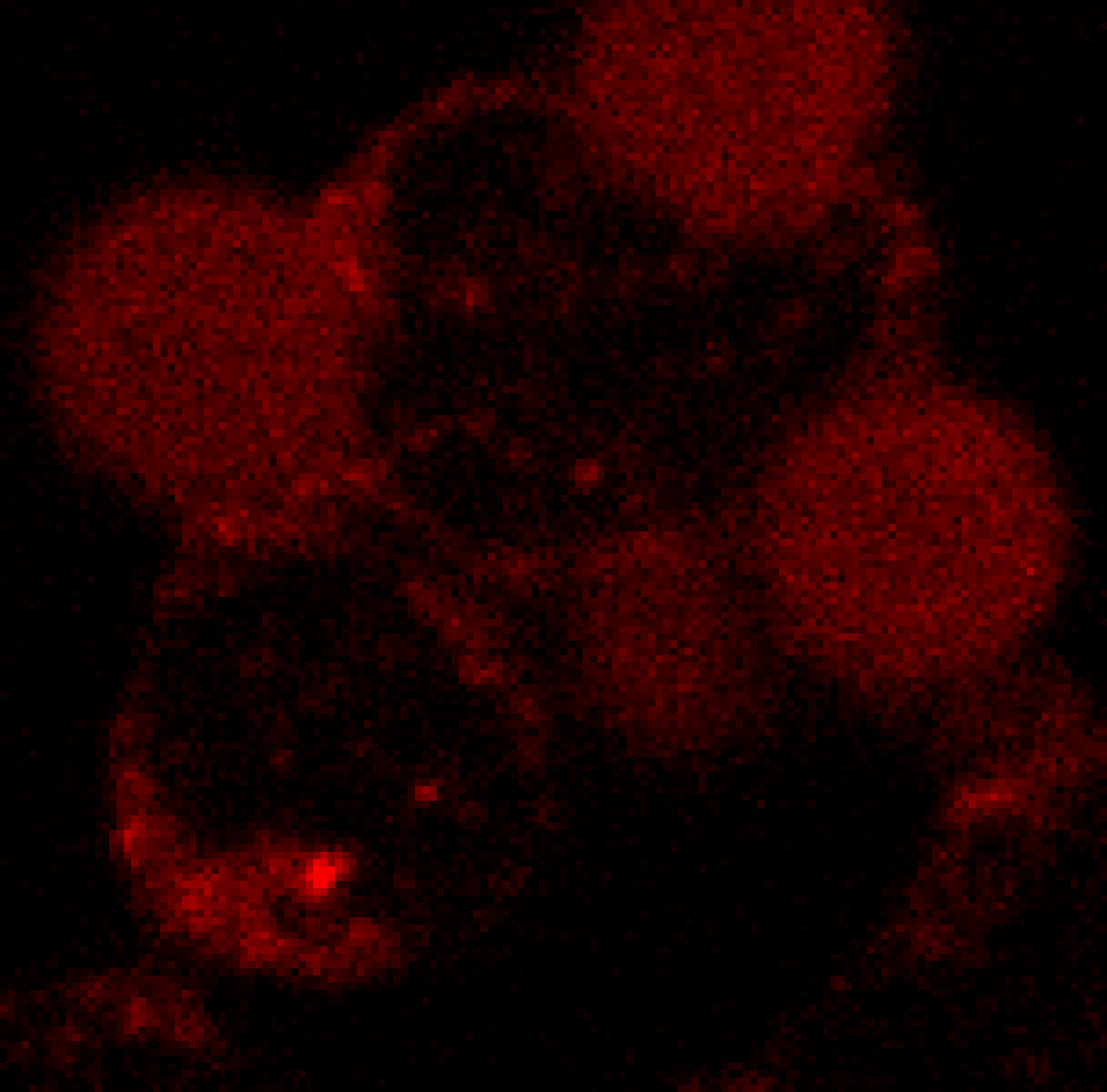

Supplement: Supplementary file 15 — Single images from Fig. 3d. [file 41590_2025_2223_MOESM15_ESM.zip › Sharma_Images_Fig3D/CsA 0.1mM NFAT1.tif]

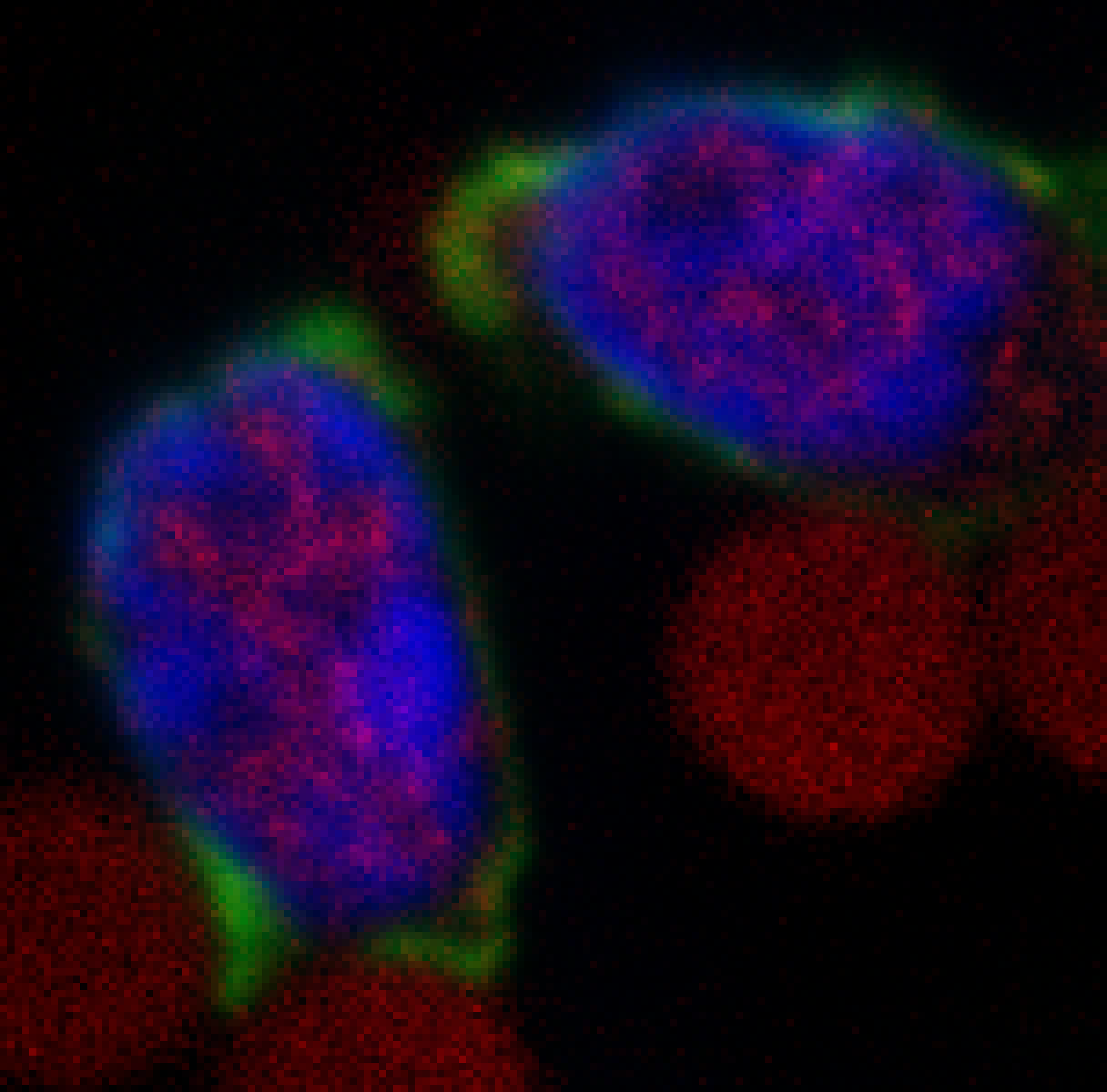

Supplement: Supplementary file 15 — Single images from Fig. 3d. [file 41590_2025_2223_MOESM15_ESM.zip › Sharma_Images_Fig3D/CONTROL 0.1mM MERGED.tif]

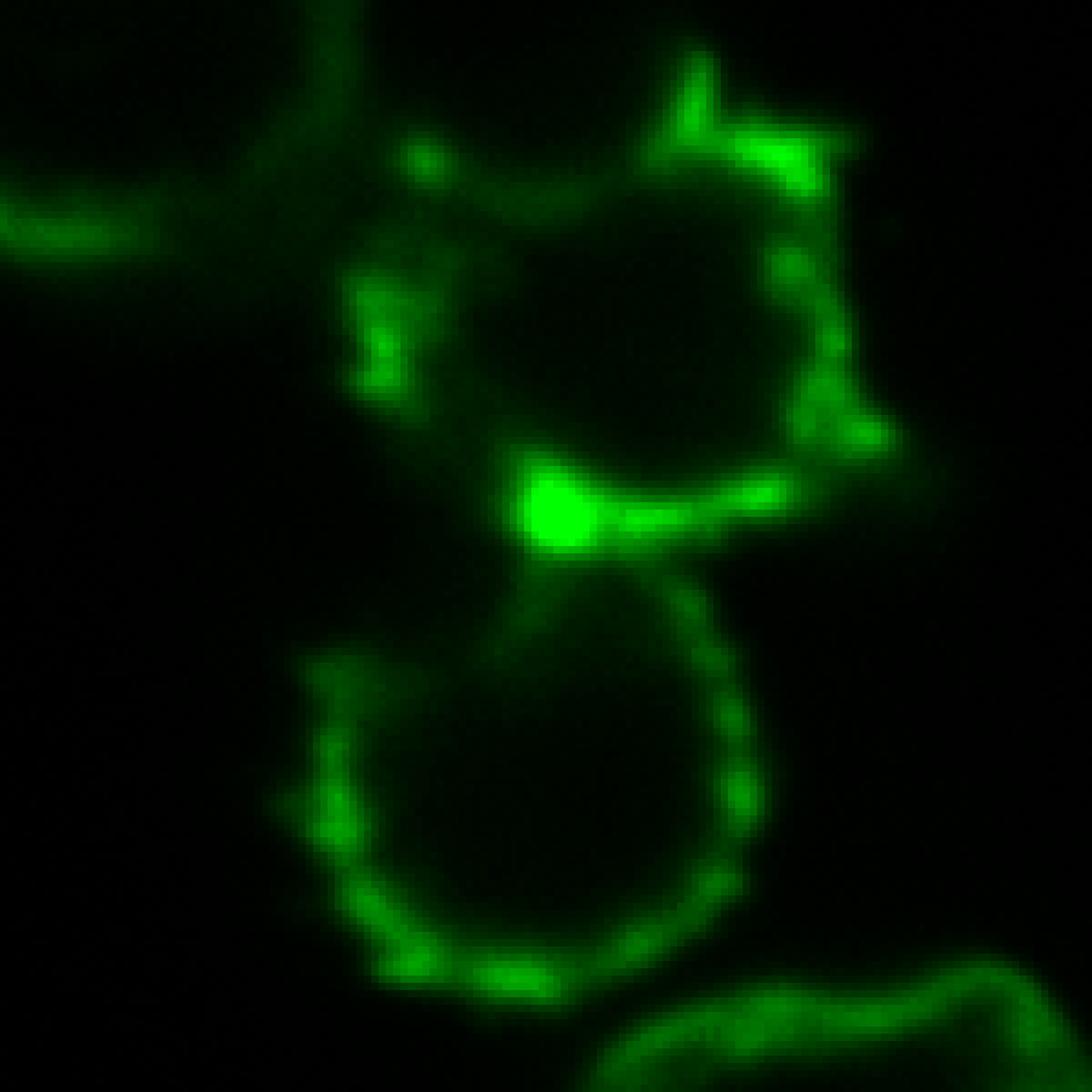

Supplement: Supplementary file 15 — Single images from Fig. 3d. [file 41590_2025_2223_MOESM15_ESM.zip › Sharma_Images_Fig3D/CsA 0.03 PHA - 2.Sub.Sub.crop.tif]

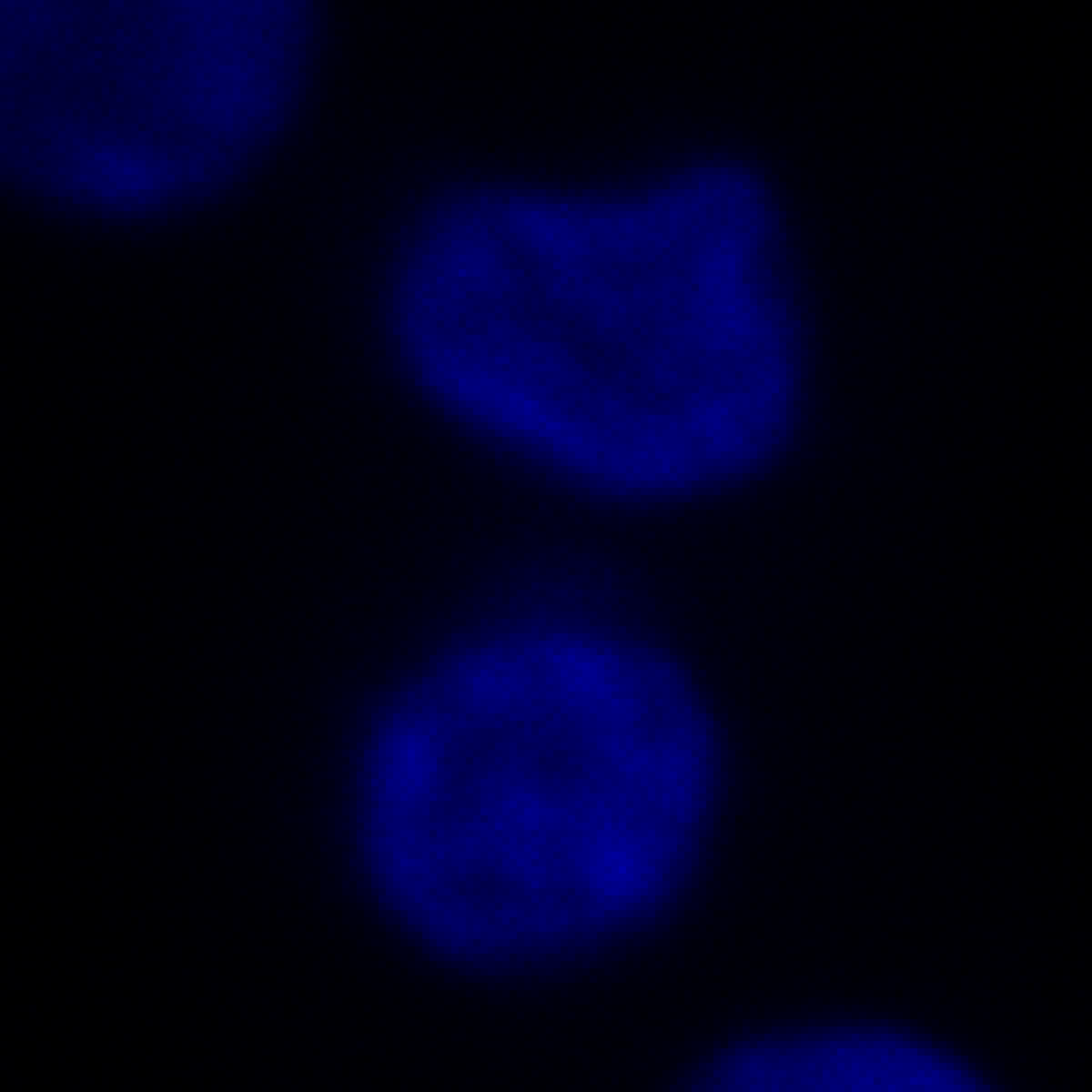

Supplement: Supplementary file 15 — Single images from Fig. 3d. [file 41590_2025_2223_MOESM15_ESM.zip › Sharma_Images_Fig3D/CsA 0.03mM HOECHST.tif]

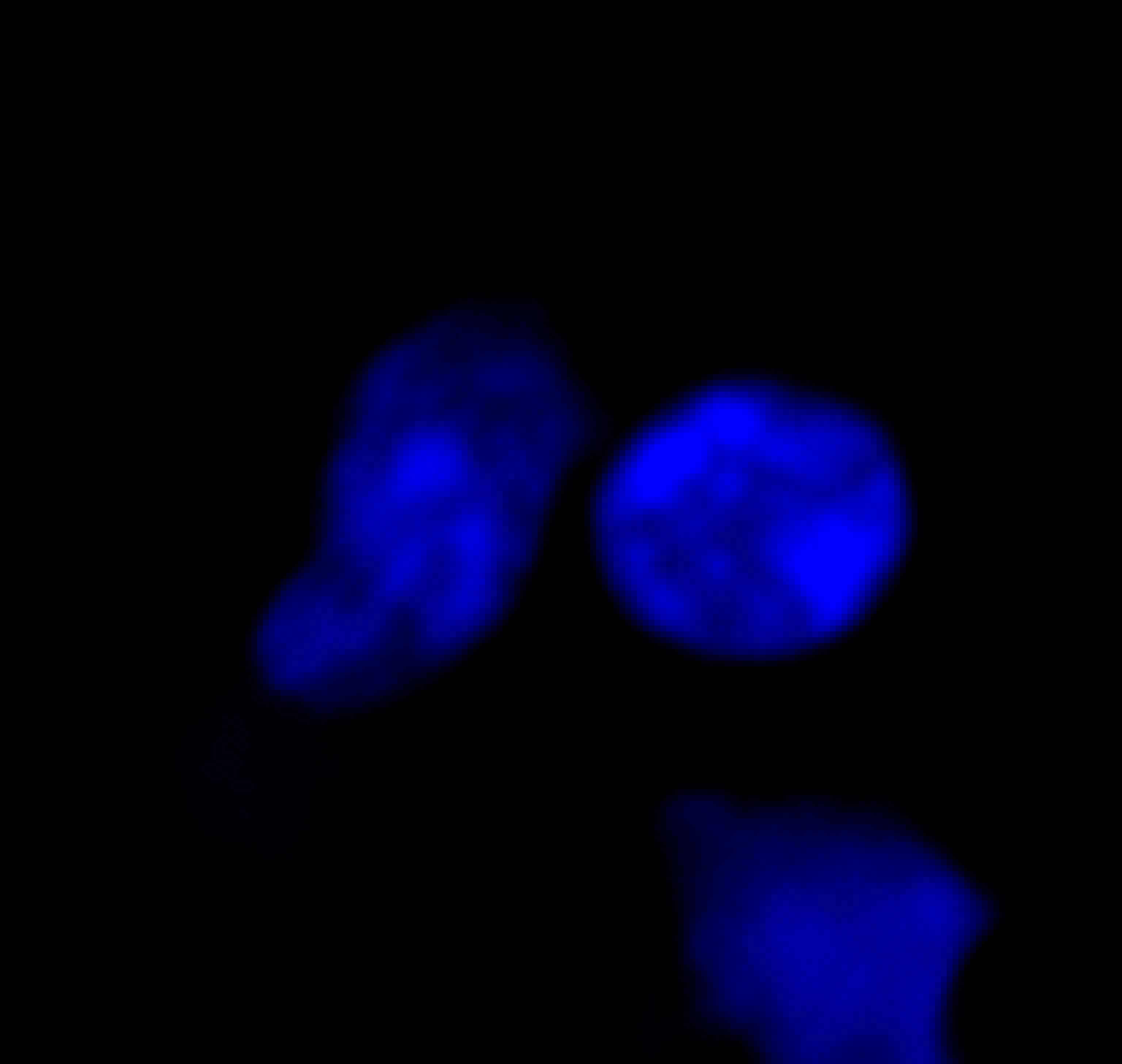

Supplement: Supplementary file 16 — Single images from Fig. 4a. [file 41590_2025_2223_MOESM16_ESM.zip › Sharma_Images_Fig4A/30MIN 0.1mM Met Hoechst.tif]

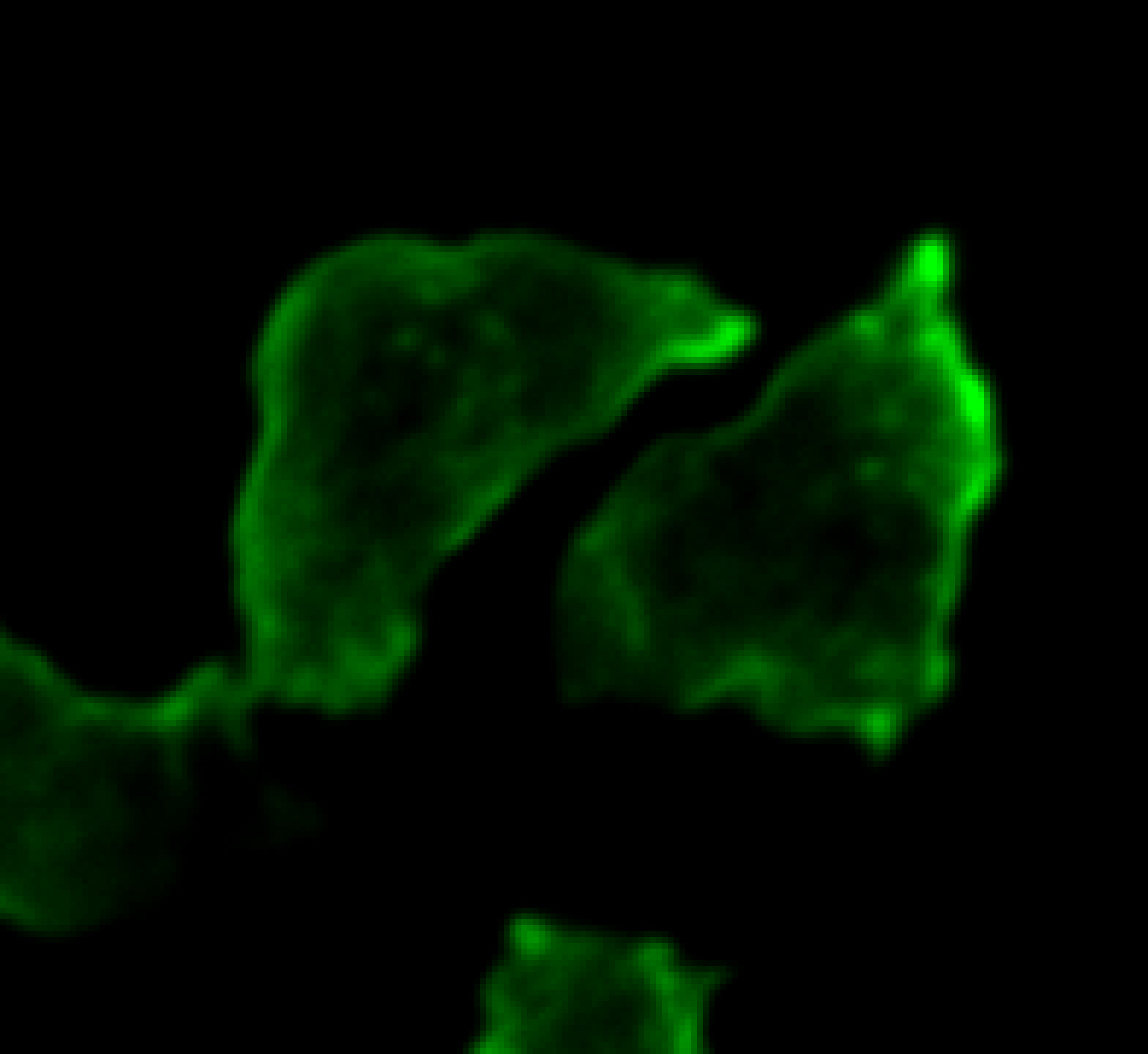

Supplement: Supplementary file 16 — Single images from Fig. 4a. [file 41590_2025_2223_MOESM16_ESM.zip › Sharma_Images_Fig4A/30MIN 0.03mM Met Phalloidin.tif]

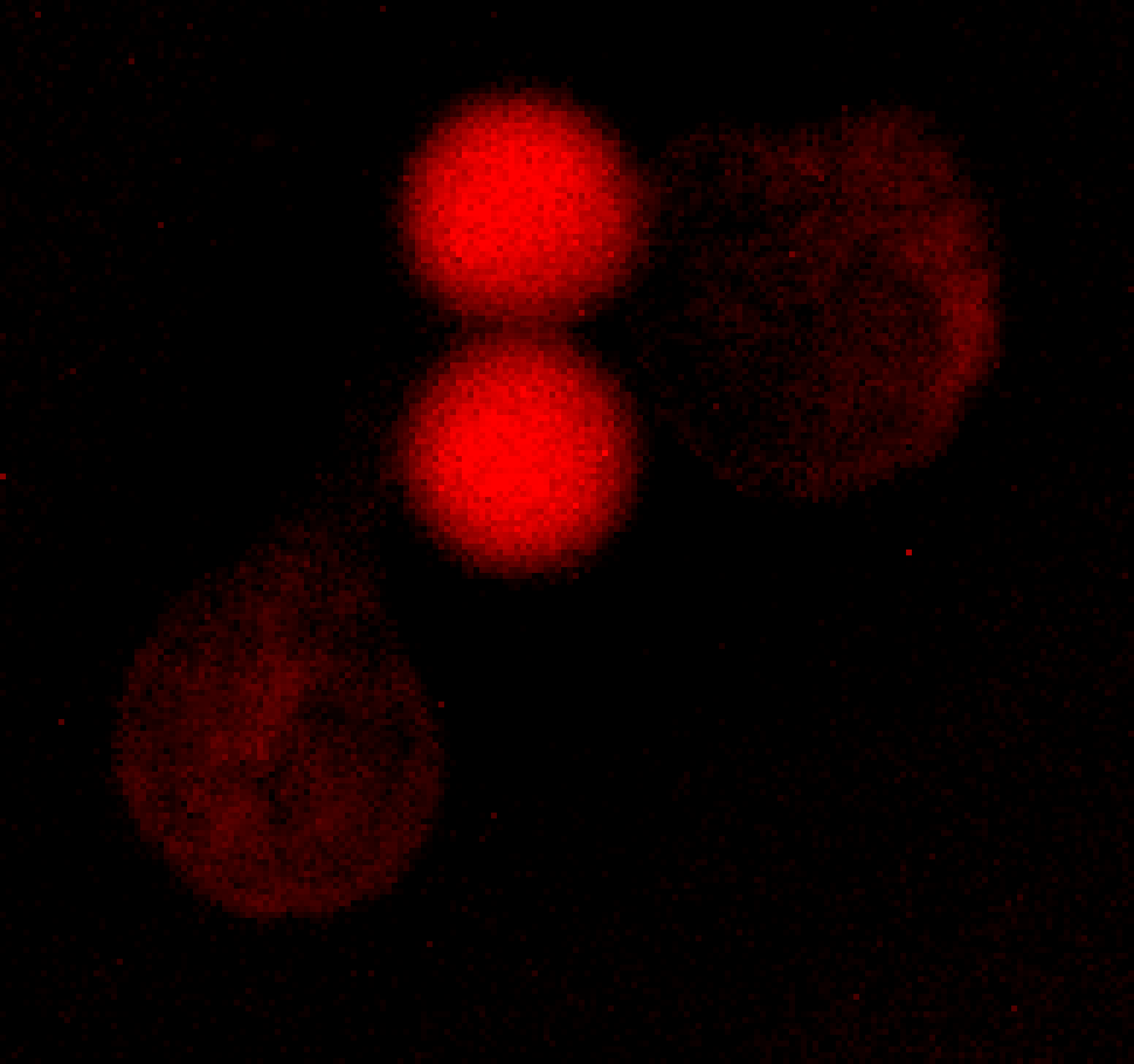

Supplement: Supplementary file 16 — Single images from Fig. 4a. [file 41590_2025_2223_MOESM16_ESM.zip › Sharma_Images_Fig4A/30MIN 0.00mM Met meARG.tif]

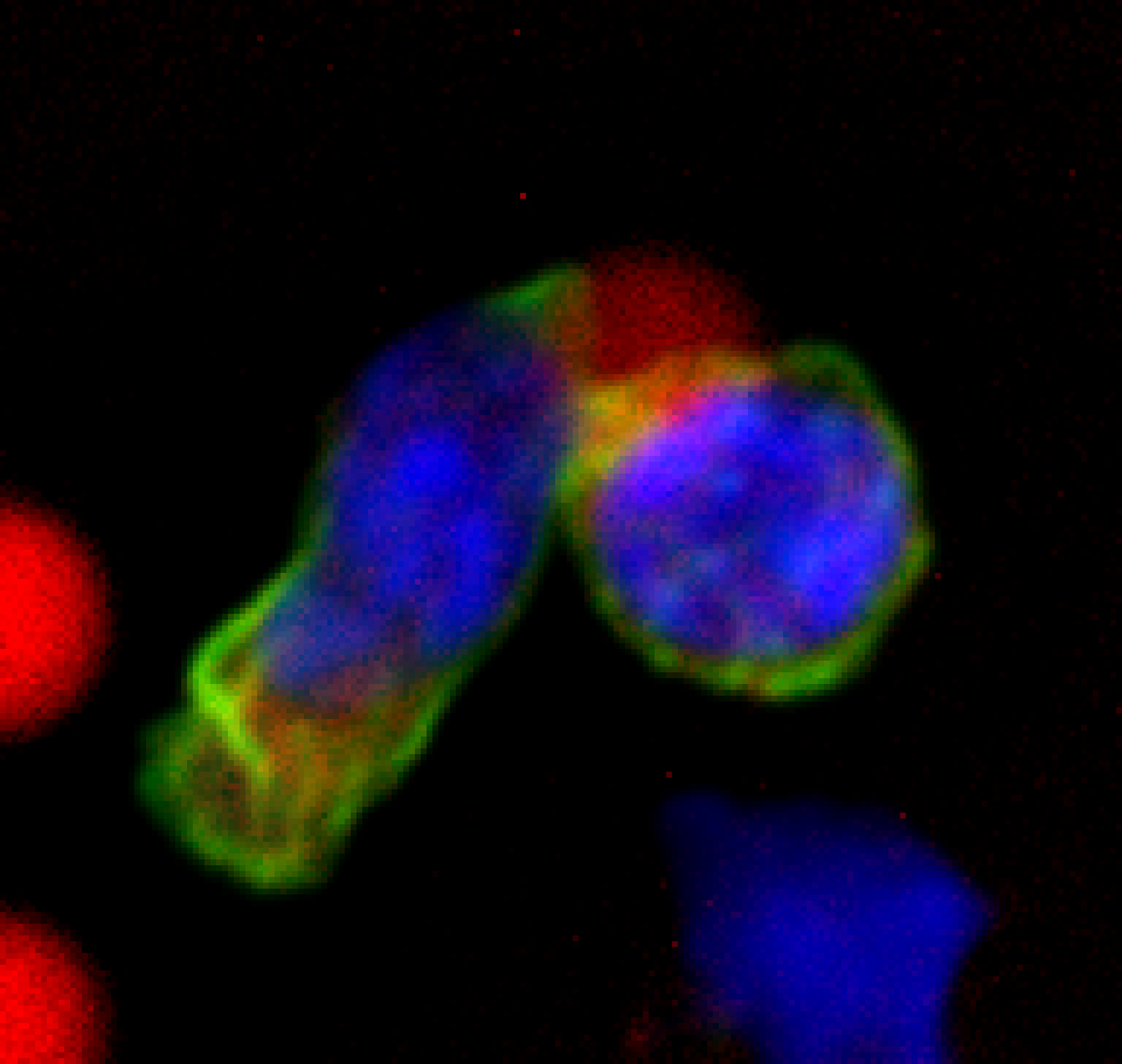

Supplement: Supplementary file 16 — Single images from Fig. 4a. [file 41590_2025_2223_MOESM16_ESM.zip › Sharma_Images_Fig4A/30MIN 0.1mM Met Merged.tif]

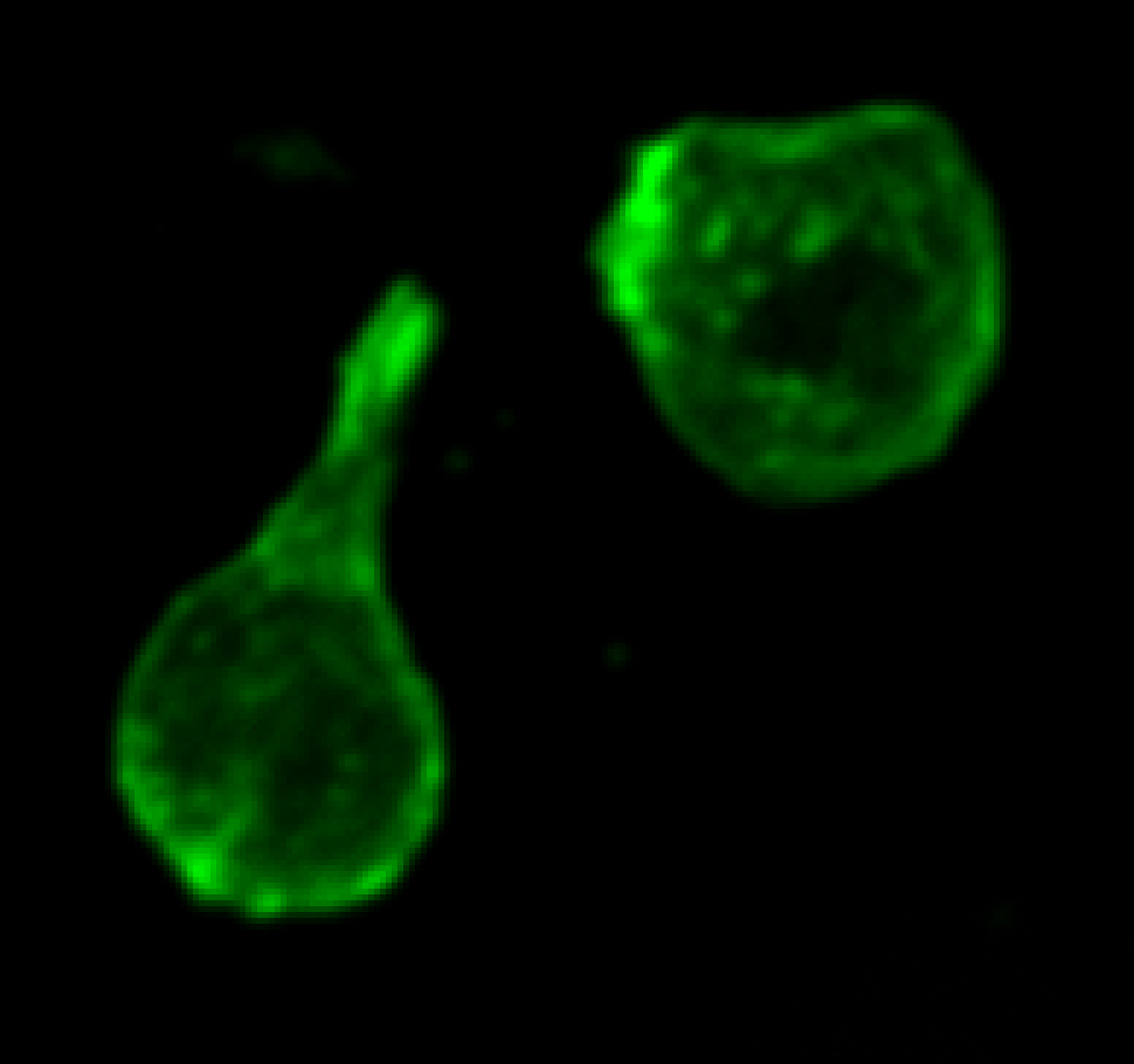

Supplement: Supplementary file 16 — Single images from Fig. 4a. [file 41590_2025_2223_MOESM16_ESM.zip › Sharma_Images_Fig4A/30MIN 0.00mM Met Phalloidin.tif]

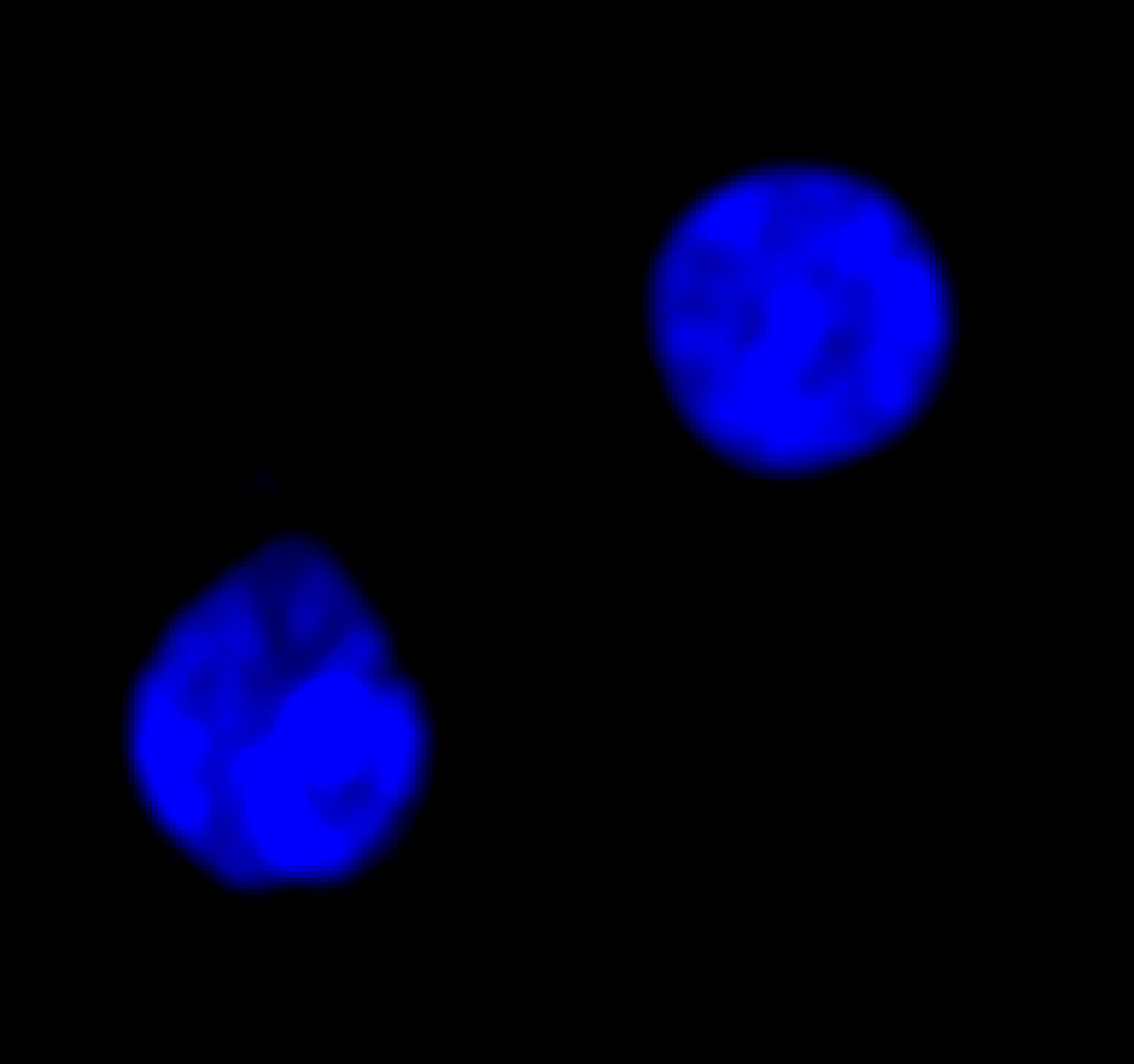

Supplement: Supplementary file 16 — Single images from Fig. 4a. [file 41590_2025_2223_MOESM16_ESM.zip › Sharma_Images_Fig4A/30MIN 0.00mM Met Hoechst.tif]

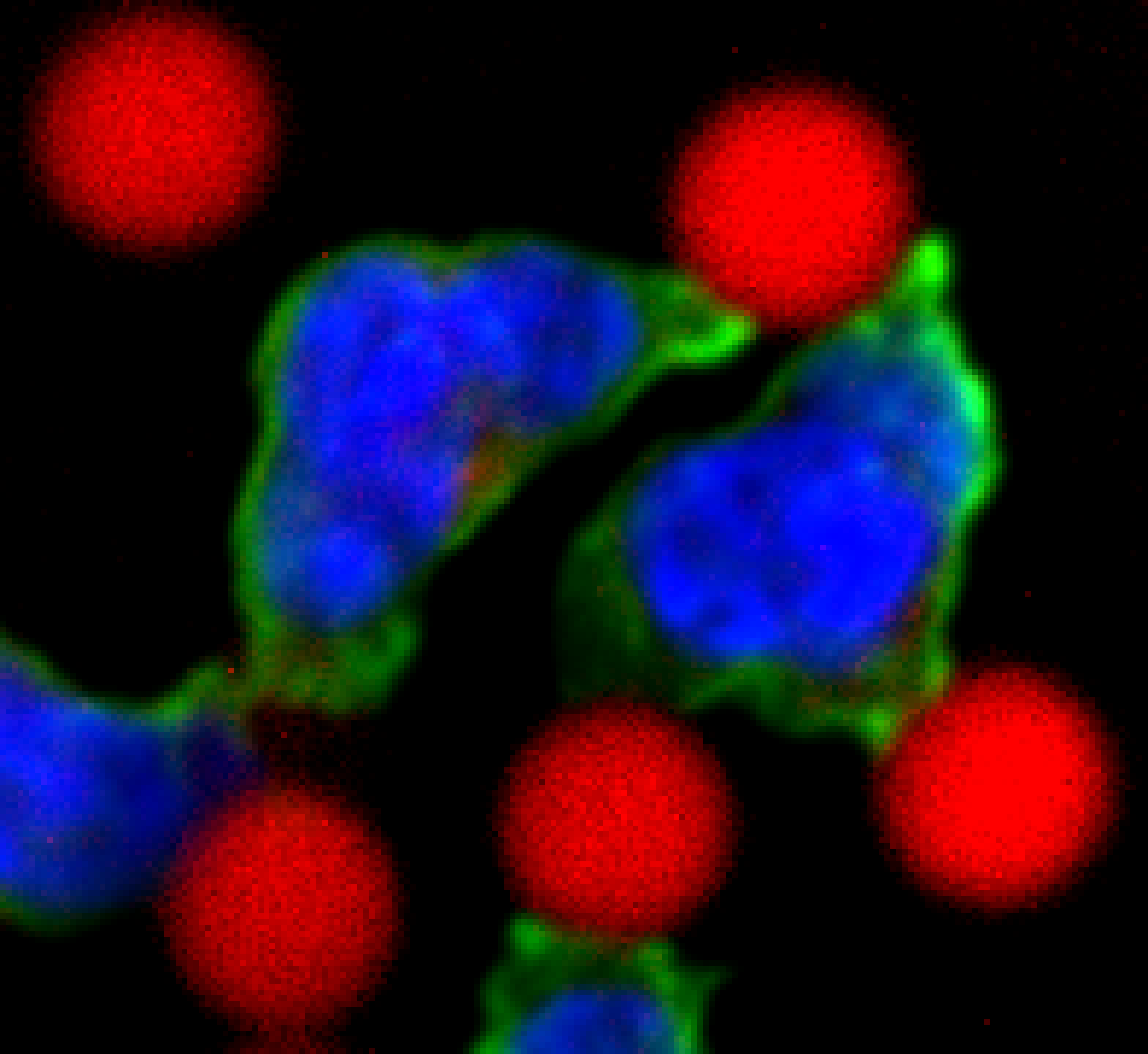

Supplement: Supplementary file 16 — Single images from Fig. 4a. [file 41590_2025_2223_MOESM16_ESM.zip › Sharma_Images_Fig4A/30MIN 0.03mM Met Merged.tif]

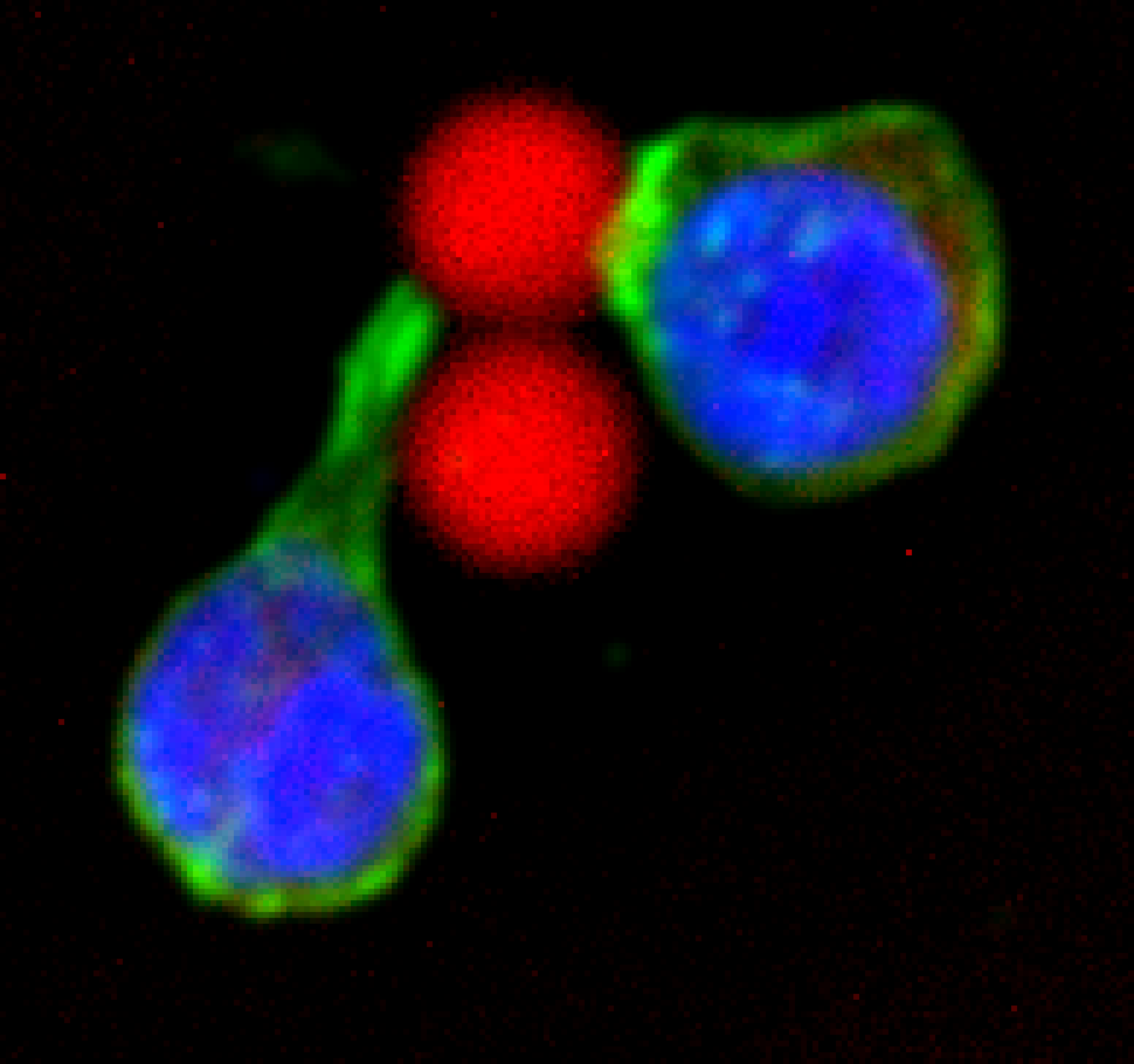

Supplement: Supplementary file 16 — Single images from Fig. 4a. [file 41590_2025_2223_MOESM16_ESM.zip › Sharma_Images_Fig4A/30MIN 0.00mM Met Merged.tif]

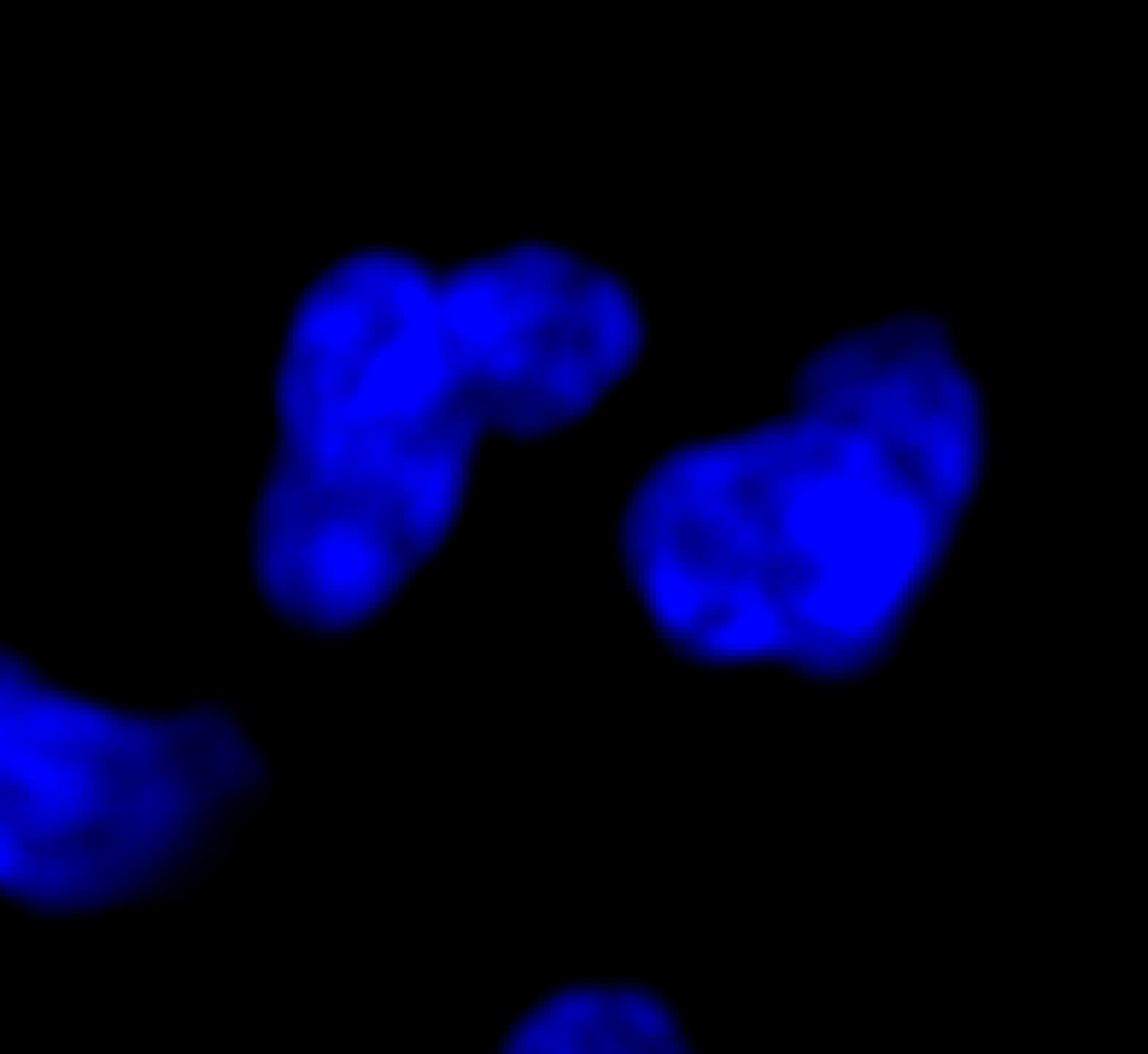

Supplement: Supplementary file 16 — Single images from Fig. 4a. [file 41590_2025_2223_MOESM16_ESM.zip › Sharma_Images_Fig4A/30MIN 0.03mM Met hoechst.tif]

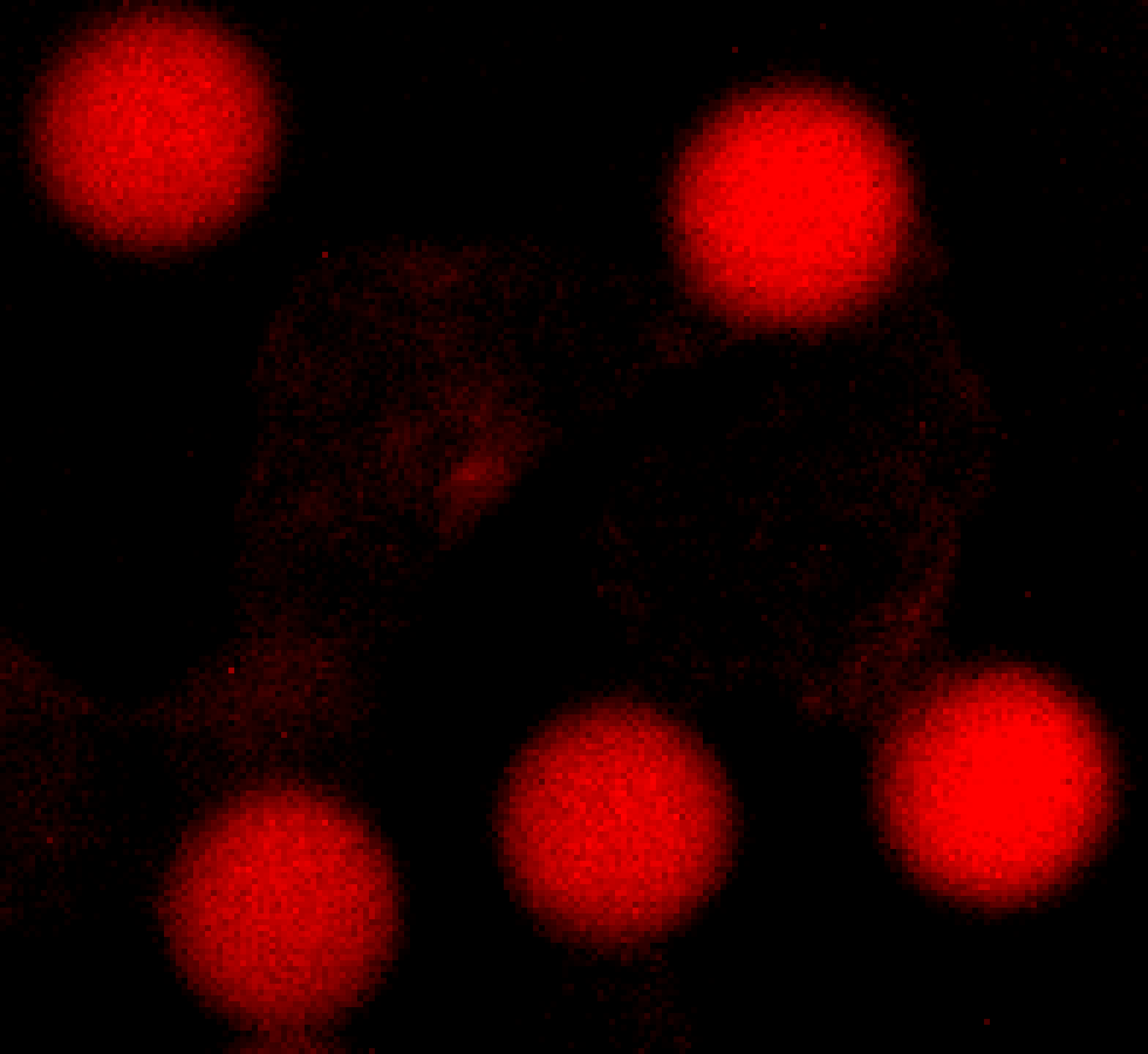

Supplement: Supplementary file 16 — Single images from Fig. 4a. [file 41590_2025_2223_MOESM16_ESM.zip › Sharma_Images_Fig4A/30MIN 0.03mM Met meARG.tif]

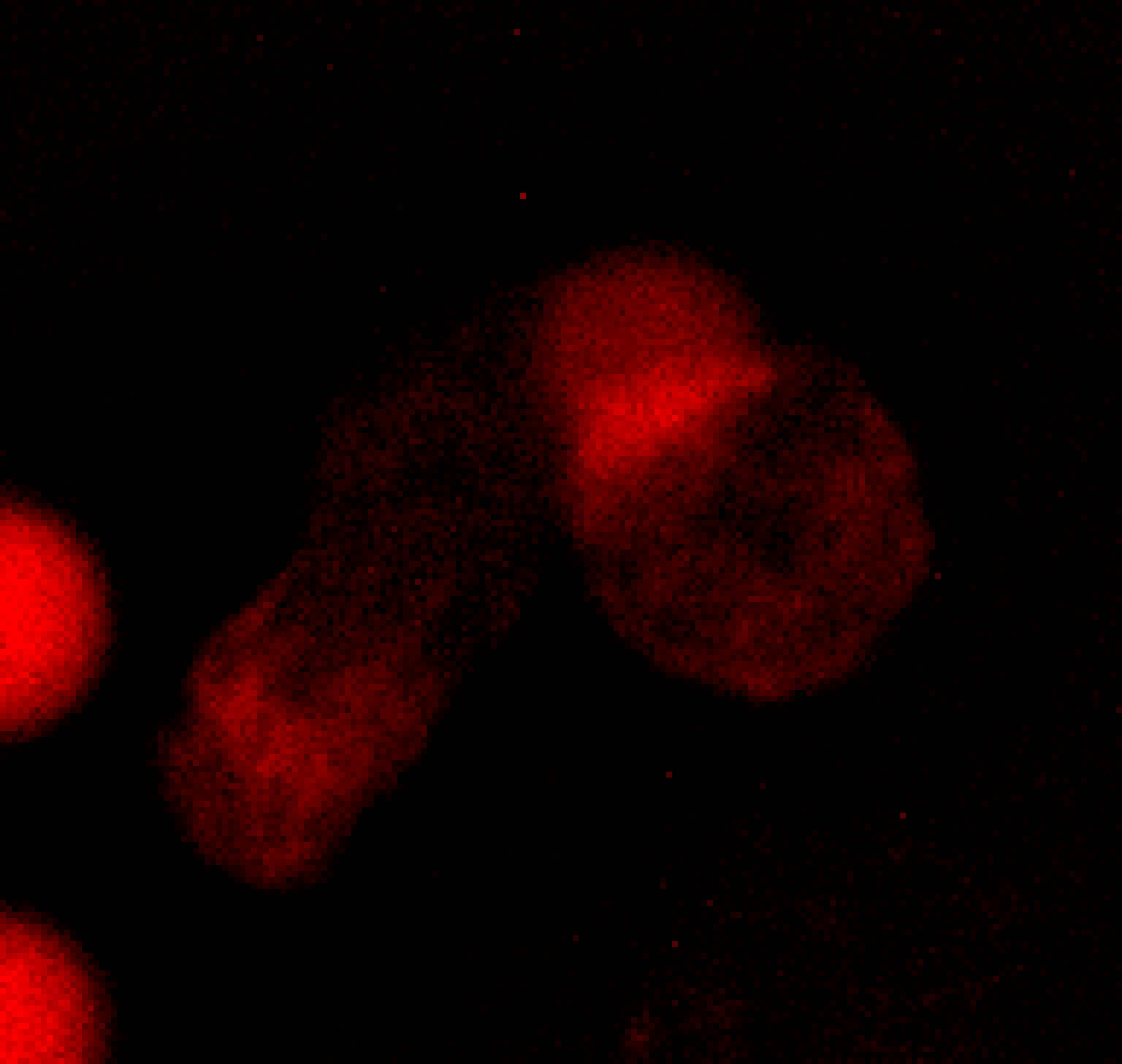

Supplement: Supplementary file 16 — Single images from Fig. 4a. [file 41590_2025_2223_MOESM16_ESM.zip › Sharma_Images_Fig4A/30MIN 0.1mM Met meARG.tif]

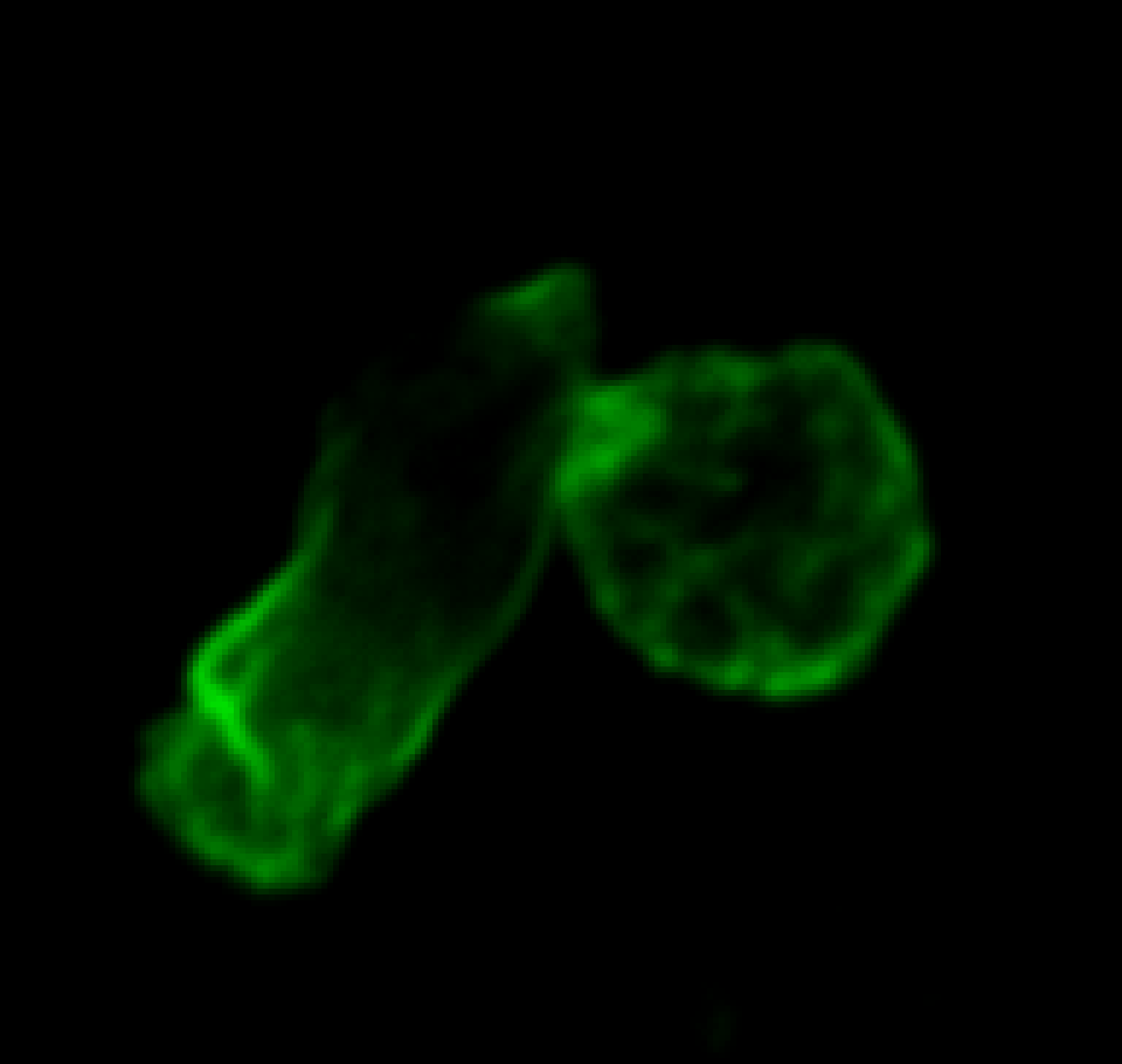

Supplement: Supplementary file 16 — Single images from Fig. 4a. [file 41590_2025_2223_MOESM16_ESM.zip › Sharma_Images_Fig4A/30MIN 0.1mM Met Phalloidin.tif]

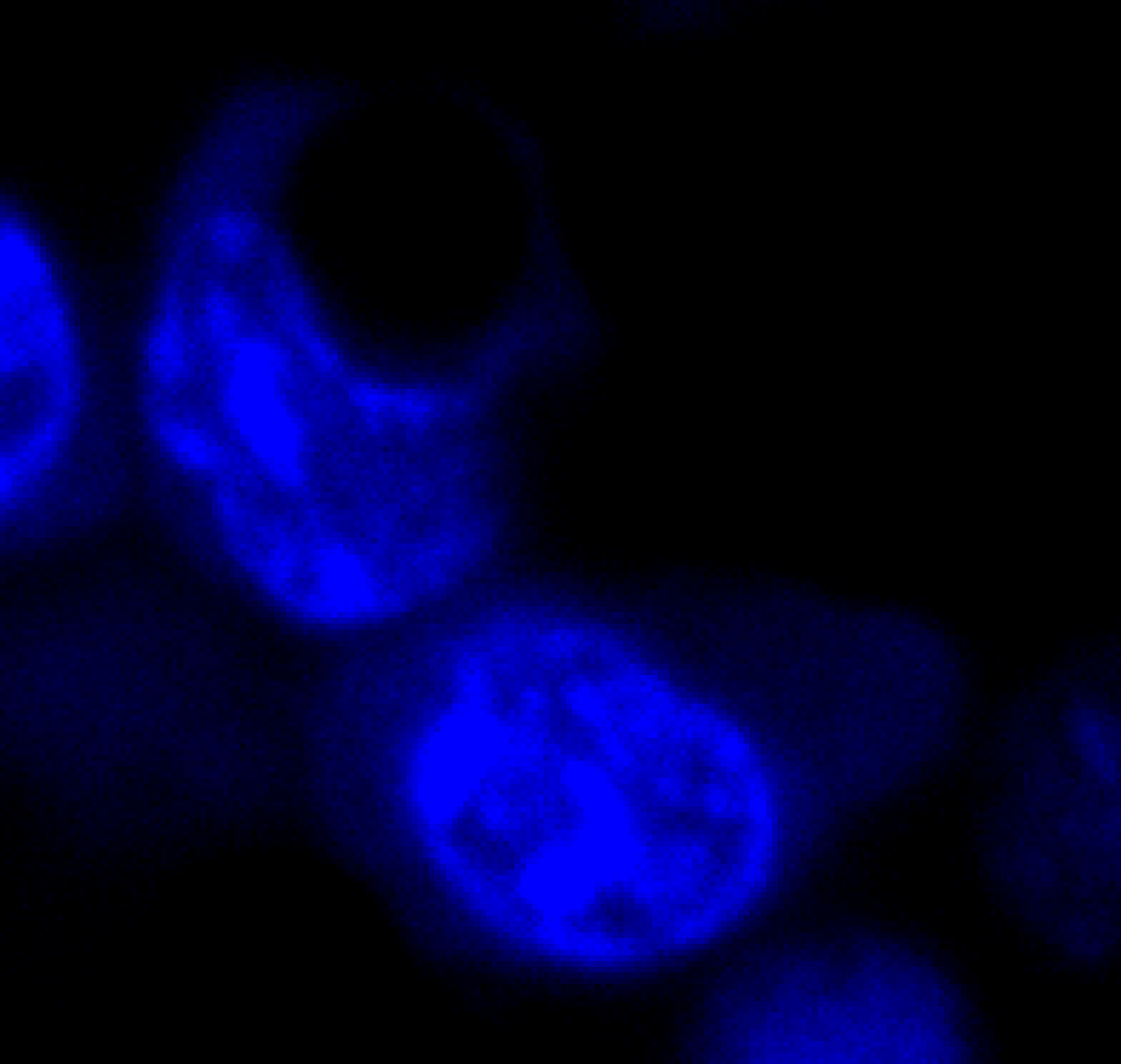

Supplement: Supplementary file 17 — Single images from Fig. 5d. [file 41590_2025_2223_MOESM17_ESM.zip › Sharma_Images_Fig5D/30MIN KCa3.1 R350A Hoechst.tif]

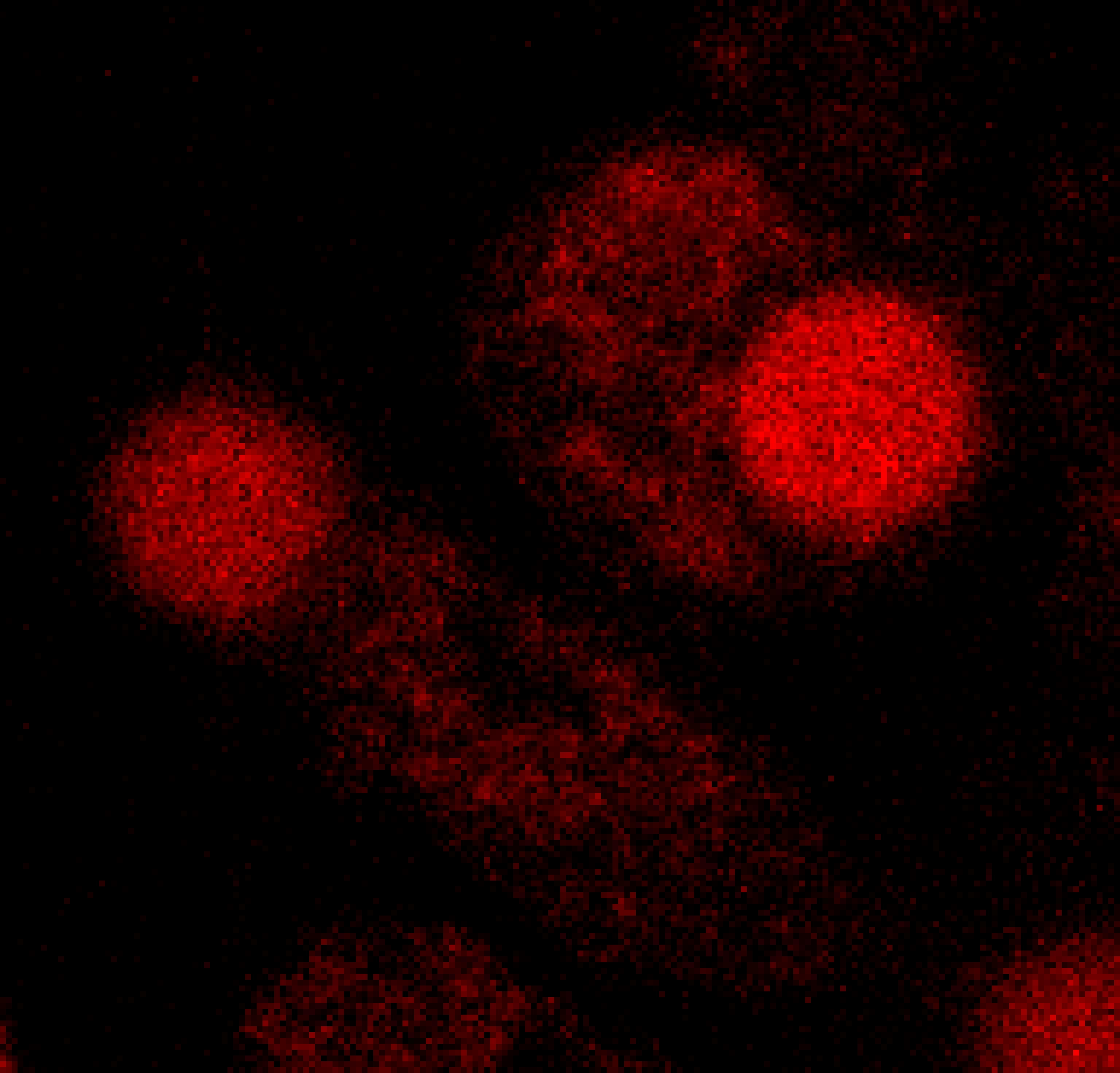

Supplement: Supplementary file 17 — Single images from Fig. 5d. [file 41590_2025_2223_MOESM17_ESM.zip › Sharma_Images_Fig5D/30MIN KCa3.1 WT NFAT1.tif]

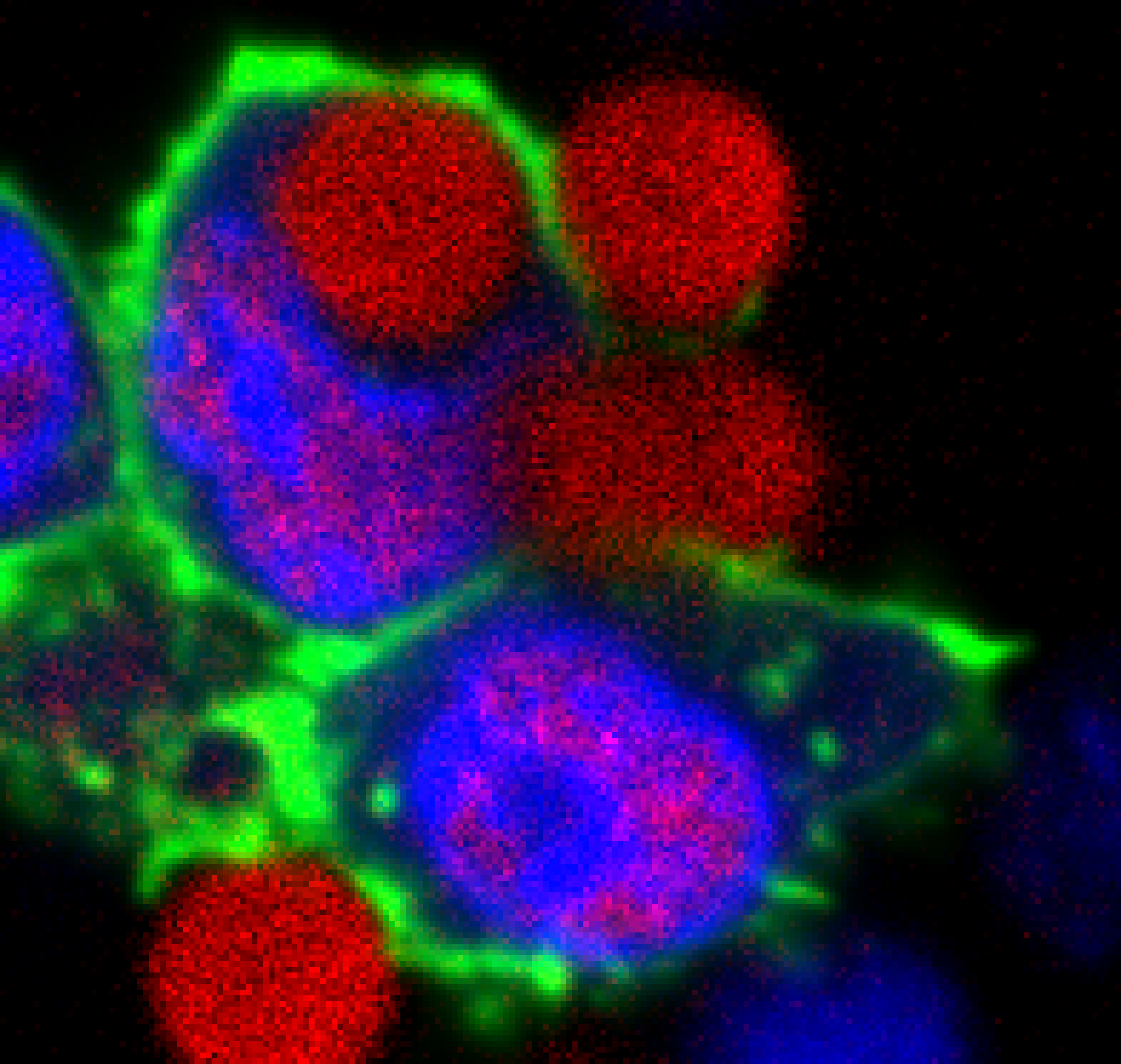

Supplement: Supplementary file 17 — Single images from Fig. 5d. [file 41590_2025_2223_MOESM17_ESM.zip › Sharma_Images_Fig5D/30MIN KCa3.1 R350A Merged.tif]

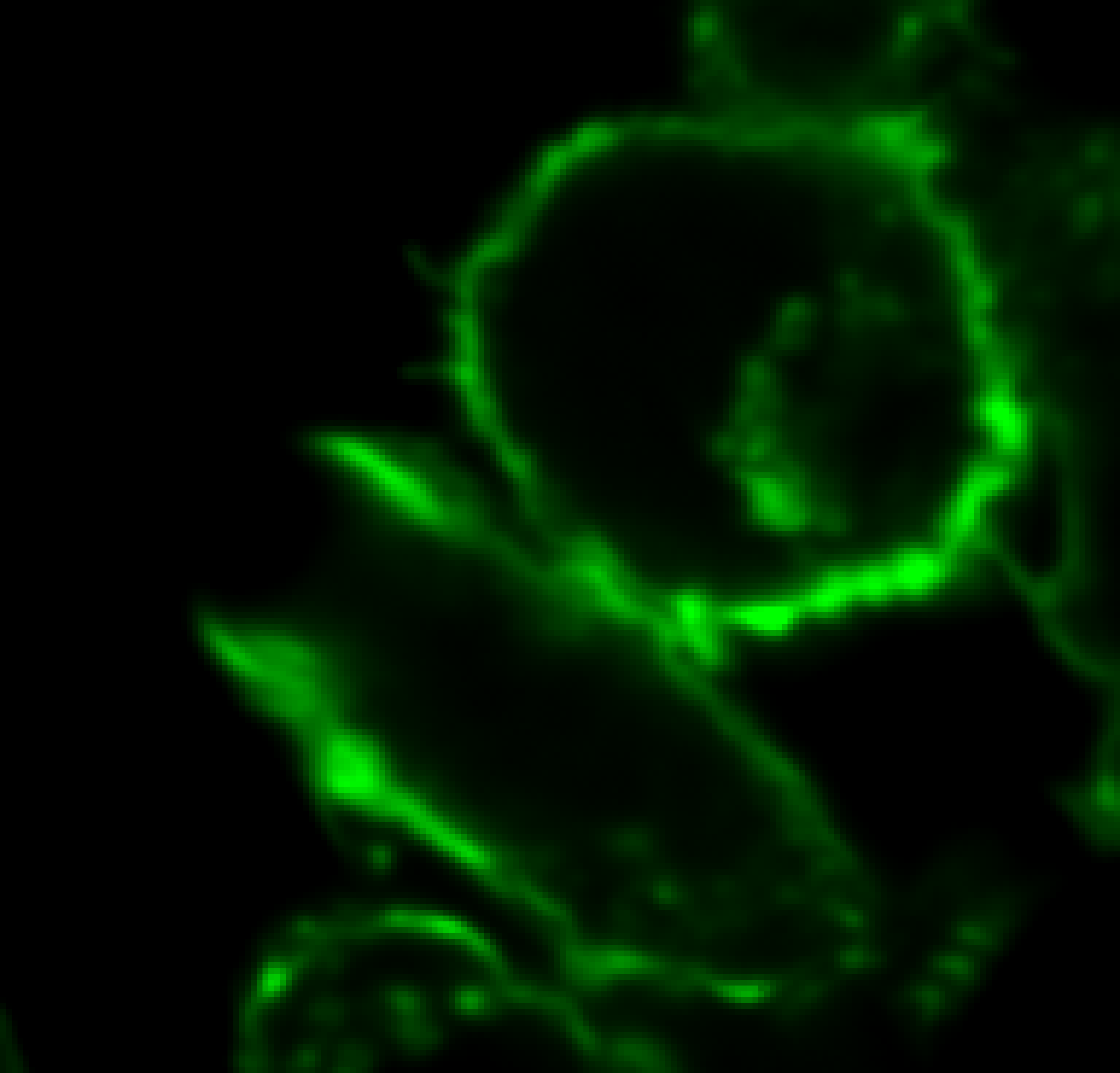

Supplement: Supplementary file 17 — Single images from Fig. 5d. [file 41590_2025_2223_MOESM17_ESM.zip › Sharma_Images_Fig5D/30MIN KCa3.1 WT Phalloidin.tif]

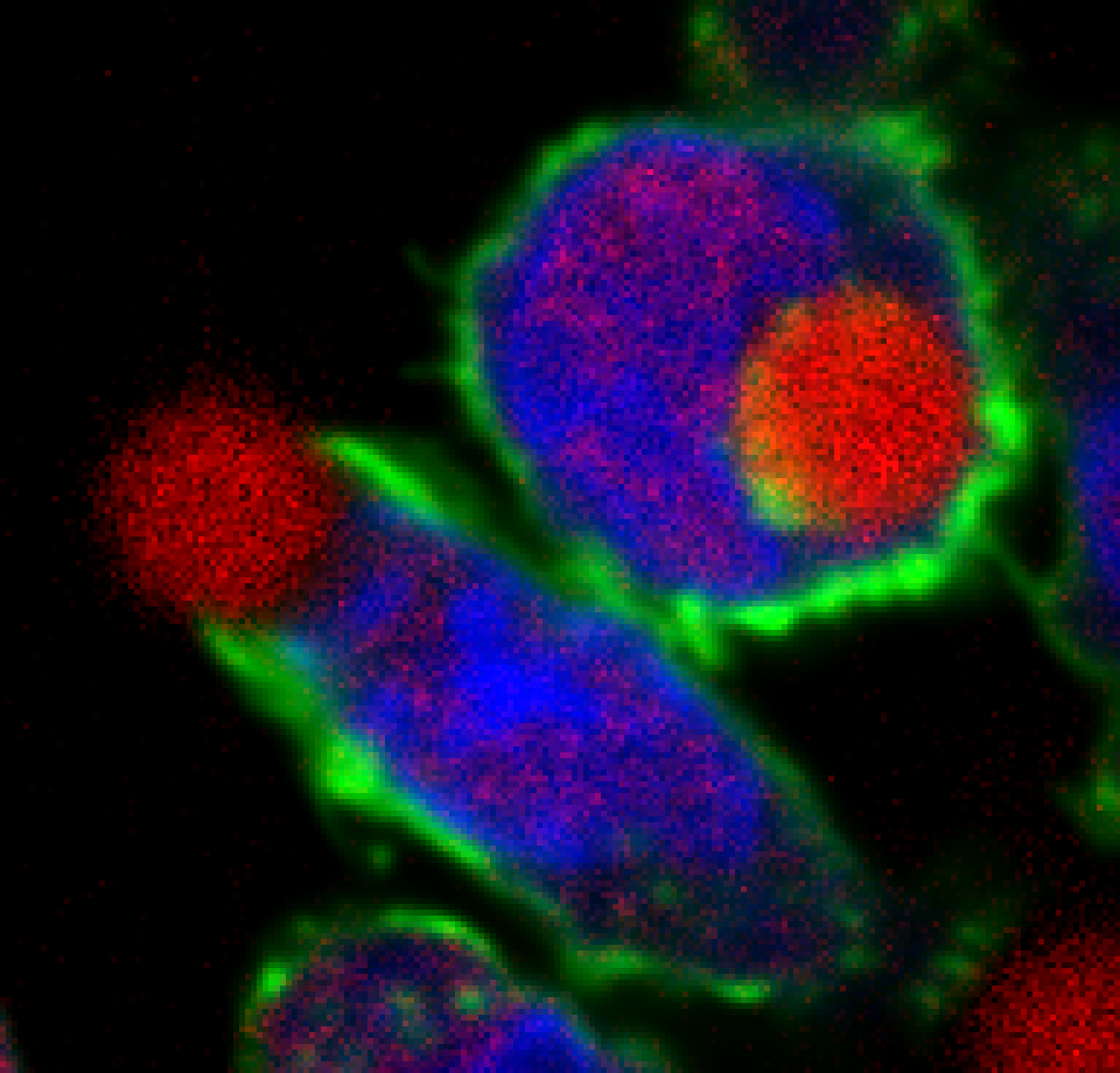

Supplement: Supplementary file 17 — Single images from Fig. 5d. [file 41590_2025_2223_MOESM17_ESM.zip › Sharma_Images_Fig5D/30MIN KCa3.1 WT Merged.tif]

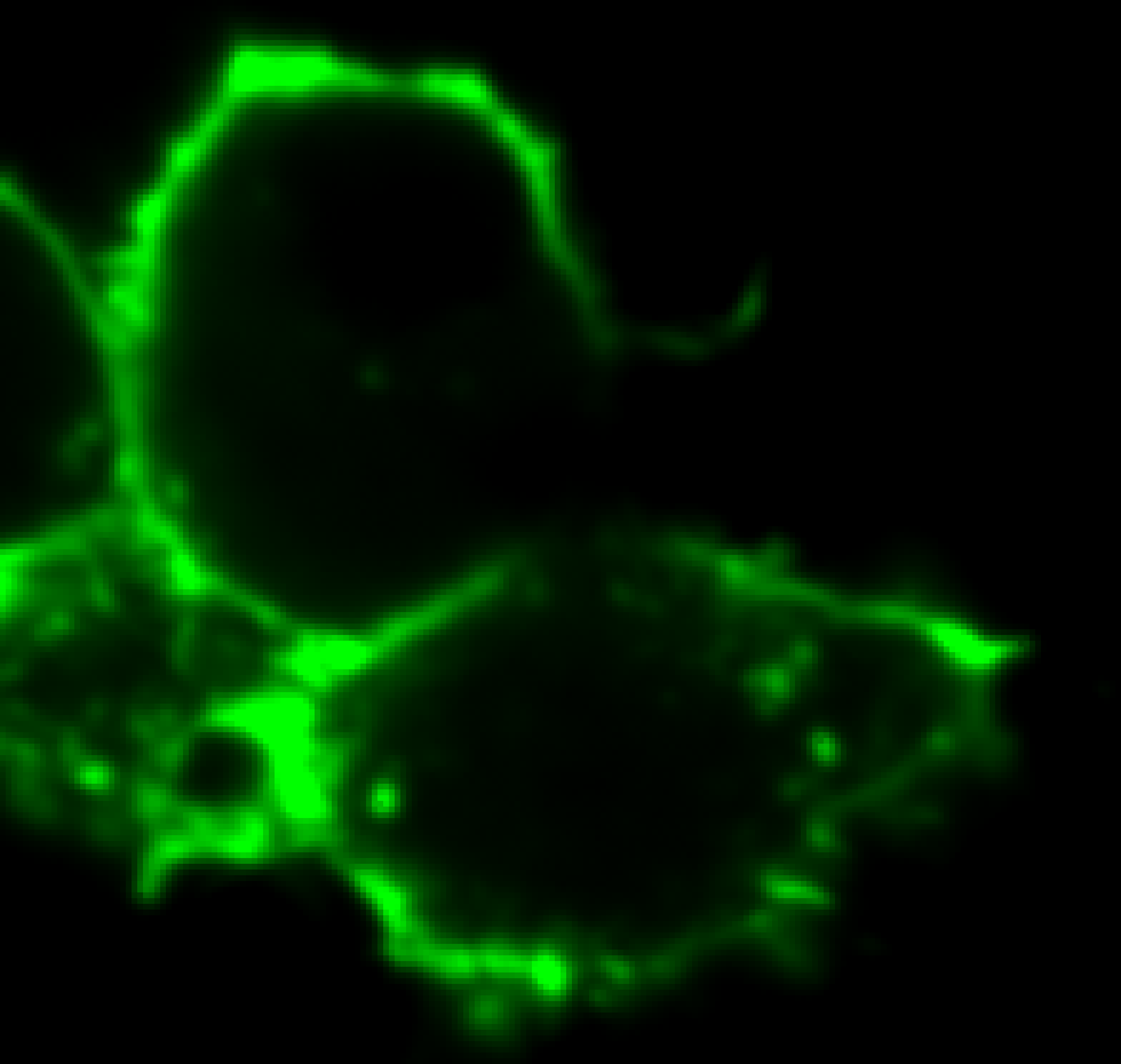

Supplement: Supplementary file 17 — Single images from Fig. 5d. [file 41590_2025_2223_MOESM17_ESM.zip › Sharma_Images_Fig5D/30MIN KCa3.1 R350A Phalloidin.tif]

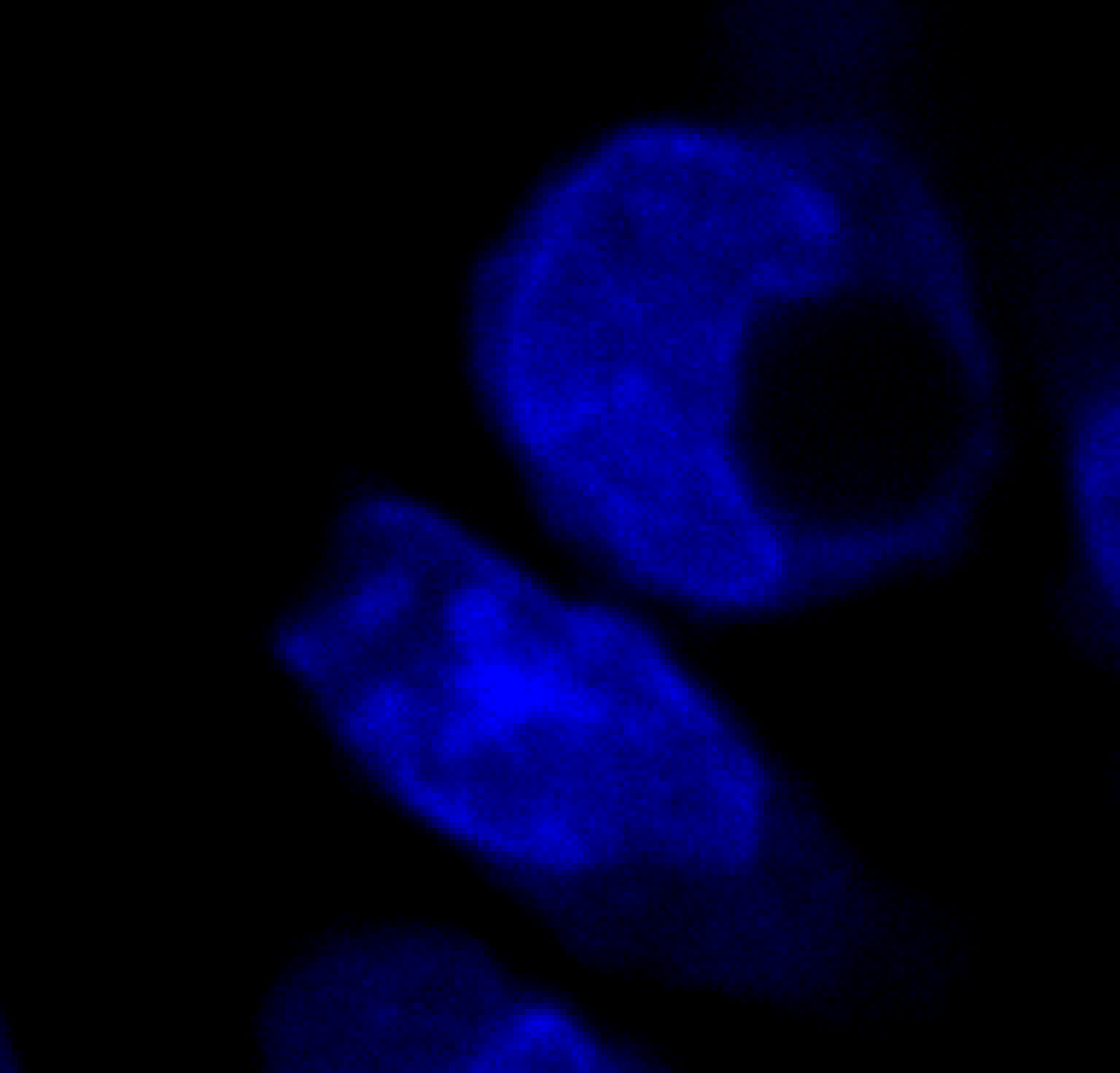

Supplement: Supplementary file 17 — Single images from Fig. 5d. [file 41590_2025_2223_MOESM17_ESM.zip › Sharma_Images_Fig5D/30MIN KCa3.1 WT Hoechst.tif]

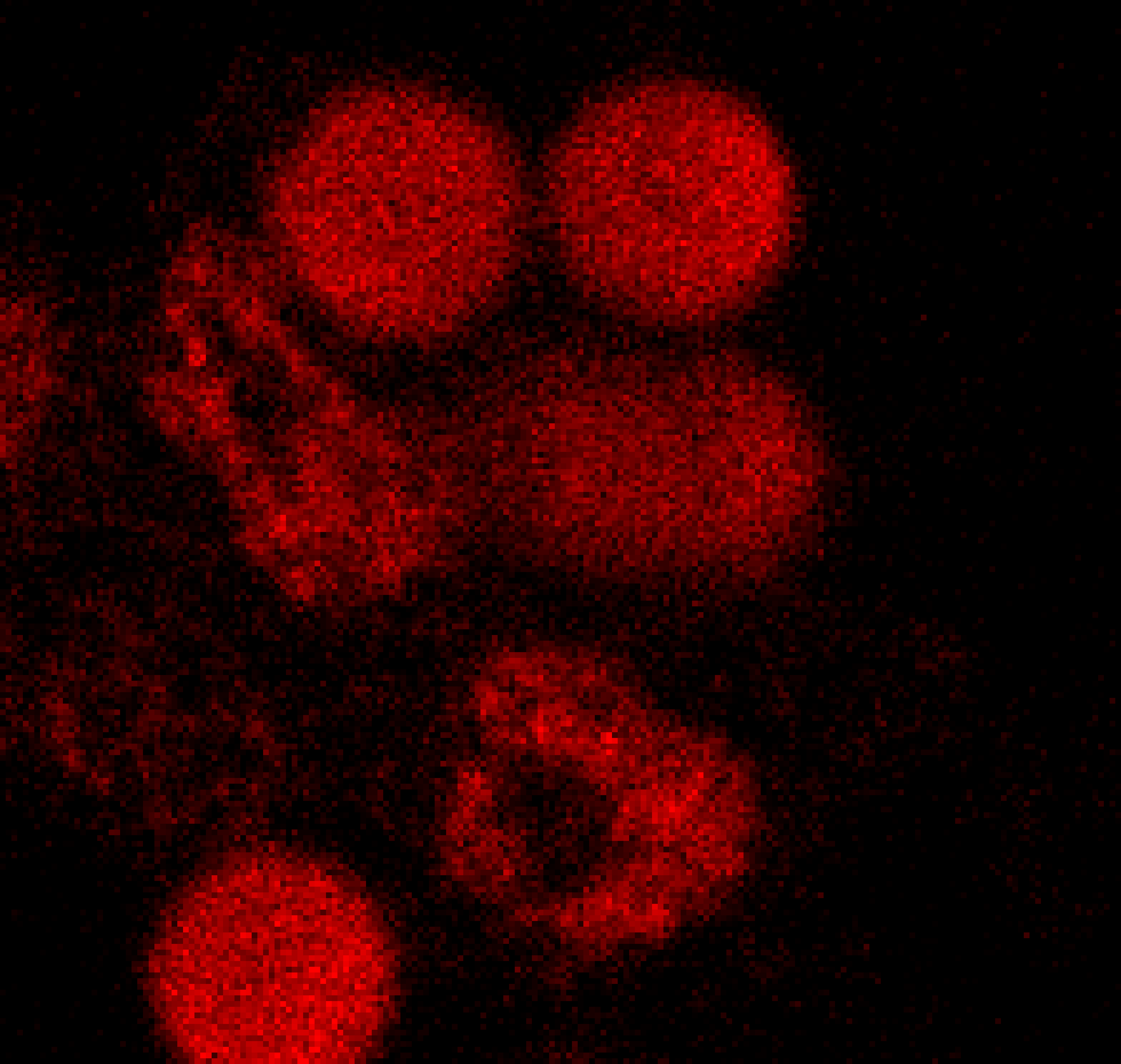

Supplement: Supplementary file 17 — Single images from Fig. 5d. [file 41590_2025_2223_MOESM17_ESM.zip › Sharma_Images_Fig5D/30MIN KCa3.1 R350A NFAT1.tif]

EXTENDED DATA FIG. 7C

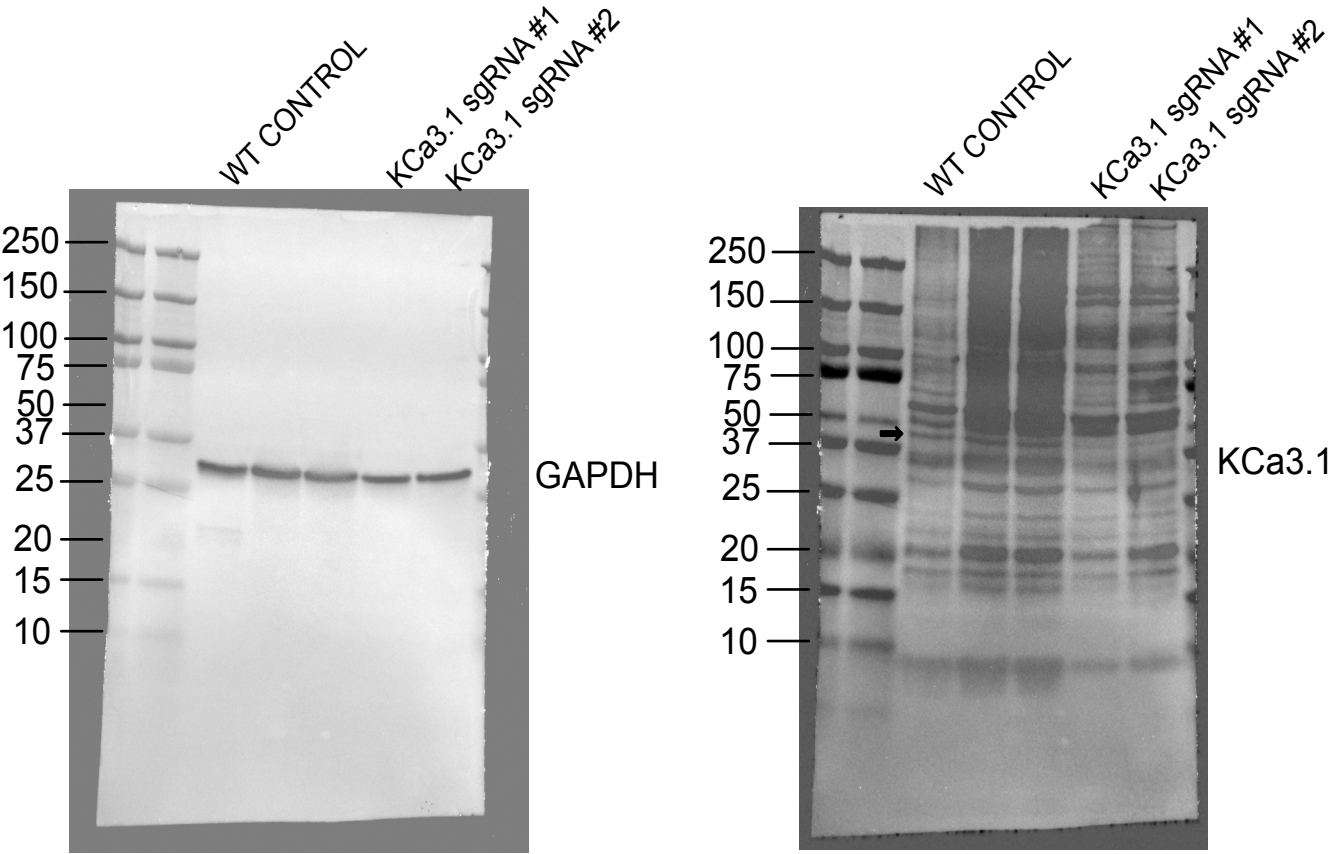

EXTENDED DATA FIG. 7D

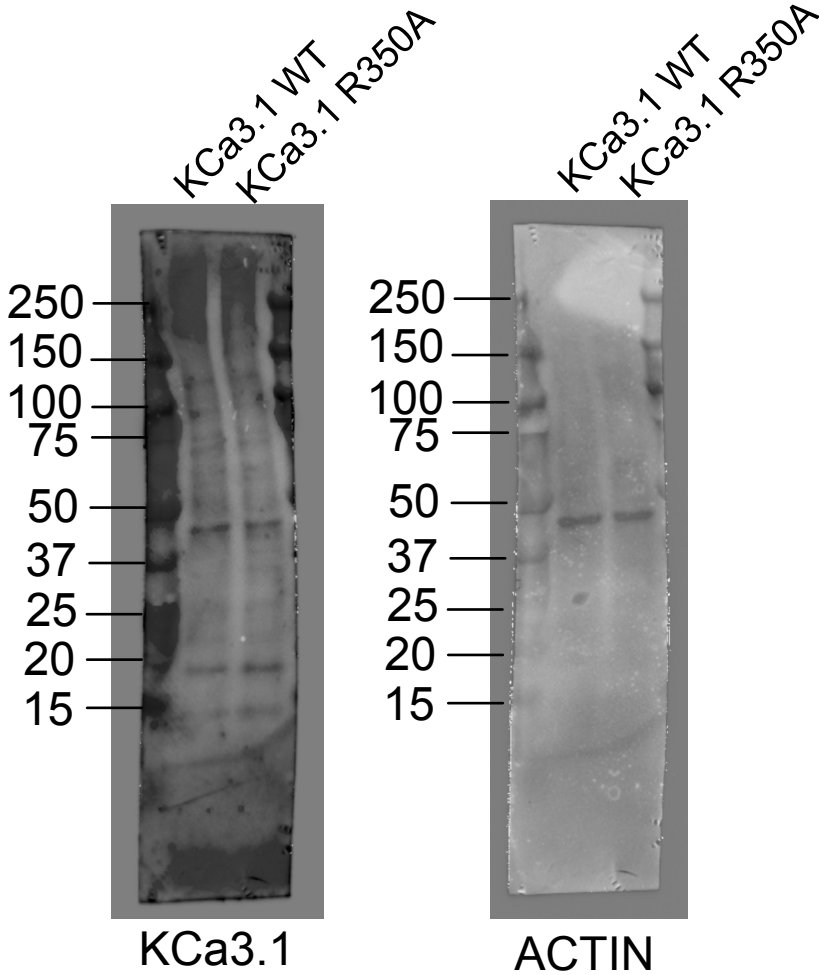

Supplement: Supplementary file 26 — Uncropped western blots. [file 41590_2025_2223_MOESM26_ESM.pdf]
